# Disrupting quorum sensing alters social interactions in *Chromobacterium violaceum*

**DOI:** 10.1038/s41522-021-00211-w

**Published:** 2021-04-22

**Authors:** Sonia Mion, Nathan Carriot, Julien Lopez, Laure Plener, Annick Ortalo-Magné, Eric Chabrière, Gérald Culioli, David Daudé

**Affiliations:** 1 Aix Marseille University, Institut de Recherche pour le Développement, Assistance Publique - Hôpitaux de Marseille, Microbes Evolution Phylogeny and Infections, Institut Hospitalo-Universitaire-Méditerranée Infection, Marseille, France; 2 Université de Toulon, MAPIEM, Toulon, France; 3 Gene&GreenTK, Marseille, France; 4 Institut Méditerranéen de Biodiversité et d’Ecologie marine et continentale, UMR CNRS-IRD, Avignon Université, Aix-Marseille Université, Avignon, France

**Keywords:** Environmental microbiology, Bacteriology, Bacteria

## Abstract

Quorum sensing (QS) is a communication system used by bacteria to coordinate a wide panel of biological functions in a cell density-dependent manner. The Gram-negative *Chromobacterium violaceum* has previously been shown to use an acyl-homoserine lactone (AHL)-based QS to regulate various behaviors, including the production of proteases, hydrogen cyanide, or antimicrobial compounds such as violacein. By using combined metabolomic and proteomic approaches, we demonstrated that QS modulates the production of antimicrobial and toxic compounds in *C. violaceum* ATCC 12472. We provided the first evidence of anisomycin antibiotic production by this strain as well as evidence of its regulation by QS and identified new AHLs produced by *C. violaceum* ATCC 12472. Furthermore, we demonstrated that targeting AHLs with lactonase leads to major QS disruption yielding significant molecular and phenotypic changes. These modifications resulted in drastic changes in social interactions between *C. violaceum* and a Gram-positive bacterium (*Bacillus cereus*), a yeast (*Saccharomyces cerevisiae*), immune cells (murine macrophages), and an animal model (planarian *Schmidtea mediterranea*). These results underscored that AHL-based QS plays a key role in the capacity of *C. violaceum* to interact with micro- and macroorganisms and that quorum quenching can affect microbial population dynamics beyond AHL-producing bacteria and Gram-negative bacteria.

## Introduction

Many proteobacteria use acyl-homoserine lactones (AHLs) to orchestrate their behavior in a cell density-dependent manner^1^. This communication system, referred to as quorum sensing (QS), plays a key role in bacterial adaptation to the environment. QS is involved in many mechanisms, including biofilm formation, production of virulence factors, and biosynthesis of antimicrobial compounds, such as phenazine^2^, bactobolin^3^, or violacein^4^. Furthermore, several studies have shown that QS plays a part not only in intra- or interspecies bacterial interactions^1,5,6^ but also in interkingdom interactions^7^.

Disrupting QS has appeared as a promising way to counteract bacterial behavior in fields of application useful for human healthcare and agriculture^8^. To this end, many strategies have been considered, including inhibition of QS signals using chemical inhibitors (QSI), their sequestration by antibodies, or the use of quorum-quenching enzymes (QQE). QQE have been particularly investigated as they can be used extracellularly to interact with signaling molecules, especially AHLs. Among QQE, lactonases, and especially those issued from extreme environments, have been shown to efficiently alter social behaviors in many proteobacteria, including *Pseudomonas aeruginosa*^9,10^, *Acinetobacter baumannii*^11,12^, *Burkholderia cenocepacia*^13^, and *Chromobacterium violaceum*^14^. Although most studies involving lactonases relied on the use of pure bacterial cultures, recent reports underlined that these biocatalysts may impact complex bacterial communities both in vitro and in situ^15,16^. Lactonase-mediated QS disruption was shown to change bacterial population dynamics and concomitantly to reduce biofilm formation in a recirculating bioreactor^15^ and also to reduce biocorrosion induced by microbial communities on steel surfaces immersed in a lake water^16^. Strikingly, metagenomic analyses revealed that lactonases did not only affect the proportion of AHL-using proteobacteria but also Gram-positive and -negative bacteria that are not known to use or sense AHLs. These results suggest that altering AHL-based QS with lactonases may drive global population changes although the mechanisms and molecular determinants of these alterations remain poorly understood.

Considering the abundance of QS-regulated traits, particularly those involved in the biosynthesis of antimicrobials, the use of exogenous QQE appeared to be a relevant approach to modulate bacterial interspecies competition^17^.

Here, we considered the use of the lactonase *Sso*Pox W263I, a QQE able to degrade a wide spectrum of AHLs and to affect microbial communities from soil and freshwater ecosystems, to decipher how QQ can modulate molecular mechanisms involved in social interactions^18^ in a single proteobacteria species representative from these ecosystems. The impact of the lactonase *Sso*Pox W263I on the model environmental bacterium *C. violaceum* ATCC 12472 (CV 12472)^19,20^ was thus investigated. Through a multi-omics approach, coupling metabolomics and proteomics together with phenotypic and microbiological investigations, the impact of enzymatic quenching on the strain behavior was deeply analyzed with a special focus on the production of antibiotic compounds^21^. We reported the production of newly observed AHLs and described, for the first time, the production of the antibiotic anisomycin in CV 12472. We further provided a series of experiments suggesting that *Sso*Pox W263I alters the ability of *C. violaceum* to produce QS-regulated antimicrobials and to compete with prokaryotic and eukaryotic organisms. Taken as a whole, these results indicate that disrupting QS may broadly impact interspecies interactions and microbial population dynamics.

## Results

### Lactonase *Sso*Pox W263I modulates *C. violaceum* proteome

**Fig. 1:**
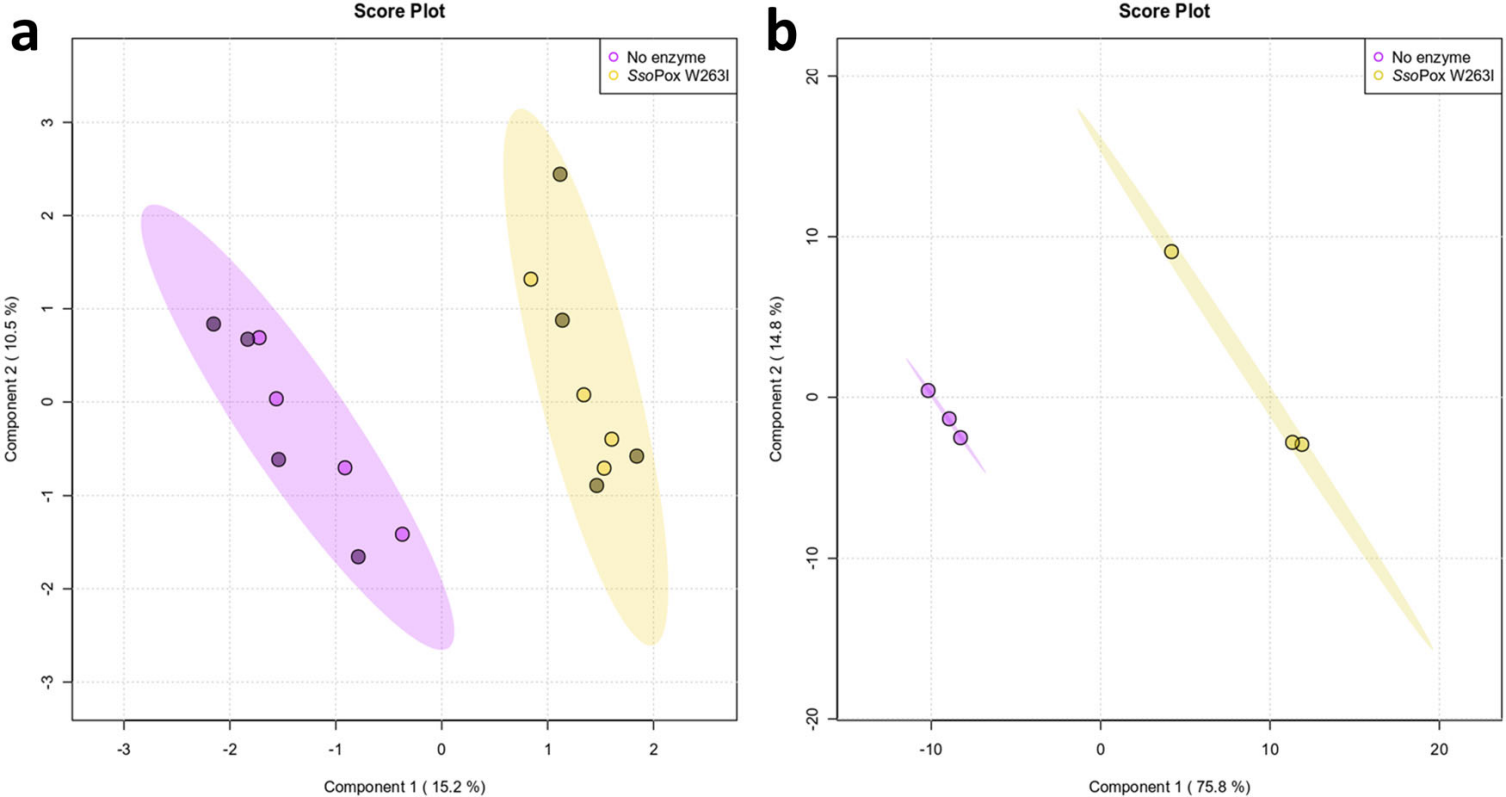
PLS-DA analysis of proteomic and metabolomic data of *C. violaceum* cultures with and without lactonase treatment. Score plots of PLS-DA analysis of proteomic (**a**) (*n* = 8: four biological replicates × two technical replicates indicated by darker point) and metabolomic data (**b**) (*n* = 3: three biological replicates) of *Sso*Pox W263I (0.5 mg ml^−1^) treated samples (yellow) and untreated ones as negative controls (purple).

Proteomes of CV 12472 with and without treatment by the QQ lactonase *Sso*Pox W263I were analyzed. The use of a statistical PLS-DA analysis revealed a significant impact of the enzyme on the proteome of CV 12472 as the control and treated groups were found distinctly separated on the first component (15.2% of the total variance) (Fig. 1a).

**Fig. 2:**
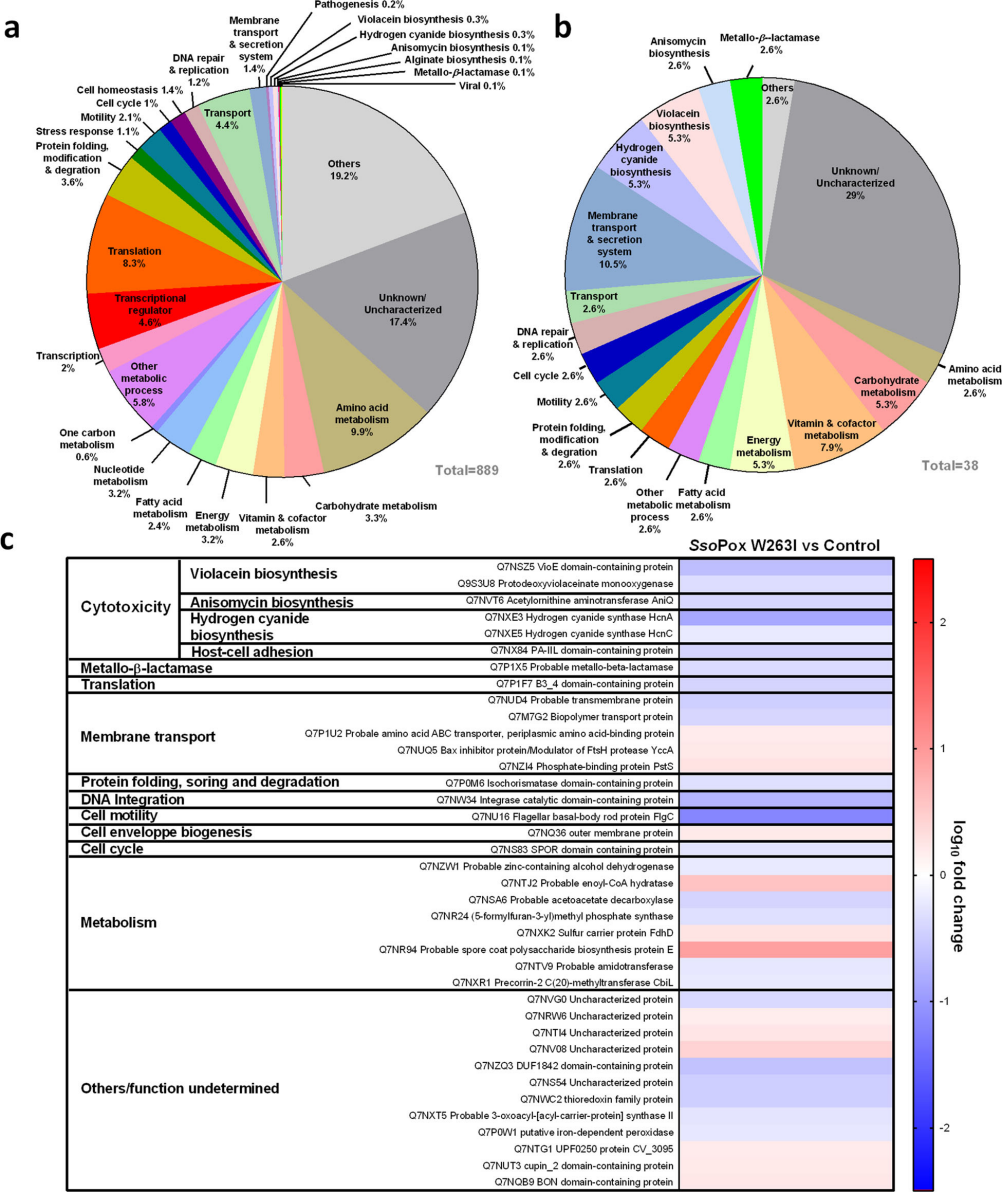
Proteome analysis of *C. violaceum* cultures with and without lactonase treatment. **a** Functional distribution of all proteins detected in *C. violaceum* samples. **b** Functional distribution of proteins significantly impacted by *Sso*Pox W263I treatment. **c** Impact of *Sso*Pox W263I treatment on *C. violaceum* protein production. Heatmap represents the log_10_ fold change in protein production of *Sso*Pox W263I-treated samples as compared to untreated ones as negative controls (selection criteria for significantly impacted proteins were *P* value <0.05 and fold change ≥1.5). Down- and upregulated protein expressions are presented in blue and red, respectively.

A total of 889 out of the 4 397 (i.e., 20.2%) proteins of CV 12472 were detected and identified (Uniprot database) (Supplementary Data 1). These 889 proteins were clustered by biological functions according to the GO database using the DAVID bioinformatic tool^22^ (Fig. 2a). This method allowed to assign of biological functions to 82.6% of this set of proteins. Proteins were mainly involved in metabolic processes (31%), followed by translation (8.3%) and transcription (6.6%).

Relative abundances of 38 proteins were found to be significantly impacted upon *Sso*Pox W263I treatment (i.e., fold change ≥1.5 and *P* value <0.05) (Fig. 2b, c) and were further clustered by biological function (Fig. 2b). Among these proteins, ten were associated with metabolic pathways, including amino acids and cofactor biosynthesis as well as the metabolism of carbohydrates, energy, and fatty acids. One protein was detected in each of the following functions: translation mechanisms, DNA repair and replication, cell cycle, and protein modification and degradation. The abundance of five proteins involved in membrane transport and secretion systems decreased upon enzymatic treatment. Interestingly, changes in proteins involved in the biosynthesis of diverse cytotoxic compounds such as hydrogen cyanide, violacein, and anisomycin were observed. These latter proteins were all downregulated by *Sso*Pox W263I treatment (Fig. 2c). Finally, twelve proteins significantly affected by the enzymatic treatment remained unknown or uncharacterized.

### *Sso*Pox W263I changes the metabolomic profile of *C. violaceum*

**Fig. 3:**
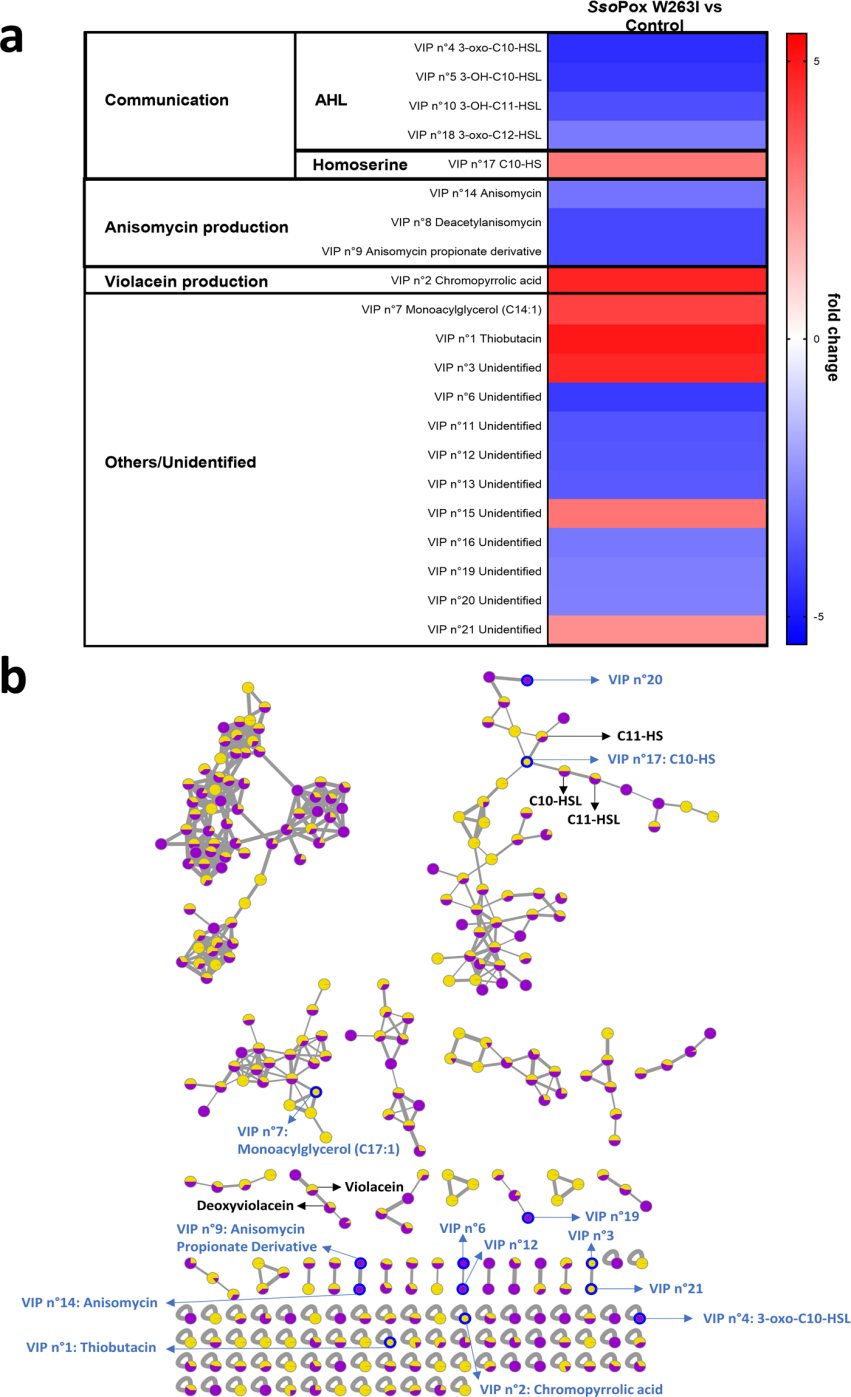
Metabolome analysis of *C. violaceum* cultures with and without lactonase treatment. **a** Impact of *Sso*Pox W263I treatment on *C. violaceum* metabolite production. Heatmap represents the fold change of the 21 most discriminating metabolites (identified by their VIP score through the PLS-DA analysis) between *Sso*Pox W263I (0.5 mg ml^−1^) treated samples and untreated ones as negative controls. Down- and upregulated metabolite expressions are presented in blue and red, respectively. **b** Molecular network of LC-MS metabolomic data and identification of molecules of interest in clusters. VIPs are circled in blue. For each metabolite, color ratio corresponds to its relative abundance in treated (yellow) and untreated (purple) samples.

To further associate proteome modifications to molecular changes, metabolomic investigations were undertaken. LC-(+)-ESI-MS metabolomic analysis enabled the identification of a primary dataset of 8 496 *m/z* features from raw LC-MS data, which was further refined and reduced down to a final dataset of 406 metabolites thanks to three filtration steps (Supplementary Table 1). The concentration of several metabolites was found to be significantly different between enzymatically treated and control samples (Fig. 3a).

PLS-DA analysis revealed two distinct groups formed by treated and control samples, separated on the first component (75.8% of the total variance) (Fig. 1b), thus indicating a significant effect of the enzymatic treatment on the metabolome of CV 12472. The PLS-DA model showed high predictive accuracy and relevance with *R*^2^ = 0.94 and *Q*^2^ = 0.84. This analysis was used to determine the variable importance in projection (VIP) score values, indicating the importance of each *m/z* feature in the discrimination between treated and control samples. Twenty-one *m/z* features with the higher VIP scores (VIP score >1.5) were selected (Fig. 3a), and the corresponding molecular formulae were determined based on accurate mass measurement, isotopic patterns, and MS/MS data analysis. In most cases, a putative identification was proposed (Supplementary Table 2).

Global Natural Products Social (GNPS) molecular networking workflow was further used to organize the whole MS/MS metabolomic dataset so as to obtain a global view of the different groups of chemical compounds (called “clusters”) containing VIPs (Fig. 3b)^23^ for annotation purposes. Such a representation allowed to gather metabolites with similar MS/MS data, and thus with putatively close chemical structures, in the same cluster. Cosine score (CS) and minimum matched fragment ions (MF) are crucial parameters to link similar metabolites together (called “nodes”) and then to build molecular networks. In this study, the use of low values of CS and MF (CS = 0.7, MF = 4) was adopted to create a maximum of links between annotated nodes and non-identified VIP nodes^24^. Some nodes observed into AHLs-, violacein-, and anisomycin-containing clusters were then identified and will be further discussed. Through this analytical procedure, more than sixty metabolites of the metabolome of CV 12472 were identified, some of which being among the VIPs that will be important in the following discussions.

### *Sso*Pox W263I hydrolyzes AHLs produced by *C. violaceum*

Both metabolome and proteome analyses revealed a significant impact of *Sso*Pox W263I on CV 12472 metabolism. To evaluate whether these discrepancies are related to QS disruption, AHLs and their potential degradation products were specifically analyzed in the cultures of CV 12472.

**Fig. 4:**
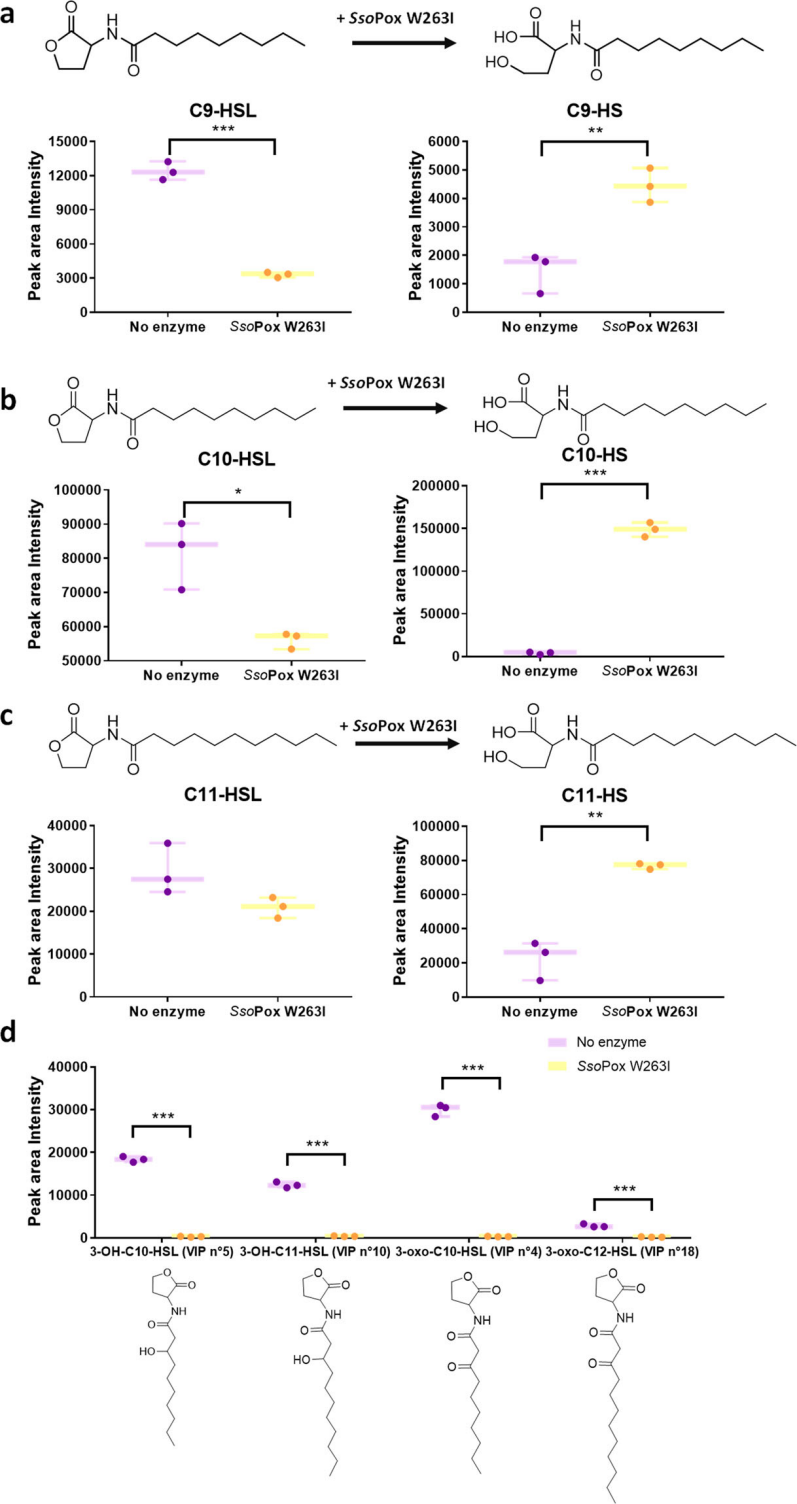
*Sso*Pox W263I impacts production of AHLs in CV 12472. Chemical structures and LC-MS peak area intensities of AHLs and their hydrolyzed form in control (purple) and treated samples (yellow). **a** C9-HSL, **b** C10-HSL, **c** C11-HSL, and **d** other AHLs. Bars represent the mean and standard deviations of peak area intensities on the corresponding LC-MS chromatograms in three biological replicates (each represented by one point). *P value <0.05, **P value <0.01, ***P value <0.001 according to Student’s *t* test.

This analysis of whole-culture extracts led to the identification of several AHLs (Fig. 4). Among these, the main AHLs observed on the chromatograms, and already known to be produced by CV 12472, were *N*-decanoyl-homoserine lactone (C10-HSL) and *N*-(3-hydroxydecanoyl)-homoserine lactone (3-OH-C10-HSL)^19–21,25^. Furthermore, non-targeted metabolomics allowed the detection and identification of AHLs reported to our knowledge, for the first time in CV 12472: *N*-nonanoyl-homoserine lactone (C9-HSL), *N*-undecanoyl-homoserine lactone (C11-HSL), *N*-(3-oxodecanoyl)-homoserine lactone (3-oxo-C10-HSL), *N*-(3-hydroxyundecanoyl)-homoserine lactone (3-OH-C11-HSL), and *N*-(3-oxododecanoyl)-homoserine lactone (3-oxo-C12-HSL). In addition, while *N*-dodecanoyl-homoserine lactone (C12-HSL) was not observed, its open form *N*-dodecanoyl-homoserine (C12-HS) was detected. Supernatant extracts obtained after removal of bacterial cells by centrifugation were also analyzed by LC-MS and allowed the detection of the same AHLs in the same proportions as those observed in the whole-culture extracts (Supplementary Fig. 1a).

The detection of diverse AHLs in CV 12472, albeit reported for the first time, is comparable to many bacterial species known to use different AHLs to regulate their QS^26^. Although C10-HSL and 3-OH-C10-HSL have been previously reported to be the main AHLs produced by CV 12472, other AHLs including 3-oxo-C10-HSL and 3-oxo-C12-HSL, have been shown to exogenously increase the production of violacein in CV 12472^19^. C11-HSL and 3-OH-C11-HSL are not common in soil bacteria and have mainly been reported in Vibrionaceae species^27,28^. C9-HSL is also rarely detected in bacteria but has already been reported in *Burkholderia cepacia* strain GG4 isolated from the ginger rhizosphere^29^, and in *Vibrio fischeri* (also called *Aliivibrio fischeri*) strains associated with the squid *Euprymna scolopes*^30^. Interestingly, C6-HSL, a molecule described as the cognate AHL used by ATCC 31532^20^, another well-described strain of *C. violaceum*, was not detected in this study, adding proof that the array of AHLs generated by *C. violaceum* appears to be strain-specific.

Although C10-HSL, and to a lesser extent 3-OH-C10-HSL and 3-oxo-C10-HSL, appeared as the main AHLs of CV 12472 whole-culture and supernatant extracts (Supplementary Fig. 1a), respective quantities of each detected AHLs and their specific impact on CV 12472 behavior are not precisely known. Further studies are necessary to determine each AHL exact concentration and contribution to QS or if some may represent inactive metabolic intermediates.

This study focused on the impact of QQE on CV 12472 behavior, to do so the impact of *Sso*Pox W263I on the relative abundance of AHLs was further analyzed. The quantity of all detected AHLs decreased significantly in the treated samples (Fig. 4). Furthermore, the open forms of C9-HSL, C10-HSL, C11-HSL, and C12-HSL, respectively, *N*-nonanoyl-homoserine (C9-HS), *N*-decanoyl-homoserine (C10-HS), *N*-undecanoyl-homoserine (C11-HS), and C12-HS, were detected in greater amounts in treated samples than in untreated ones (Fig. 4a–c). Such an observation is consistent with the previously reported mechanism of *Sso*Pox^31^ and with the catalytic performance of *Sso*Pox W263I toward the AHLs detected in this study (Supplementary Table 3) and the autoinduction mechanism described in CV 12472^21^.

These results confirmed the hydrolytic action of *Sso*Pox W263I on the AHLs produced by CV 12472 and highlighted its QQ potential.

### Lactonase drastically decreases the production of known QS-associated factors

**Fig. 5:**
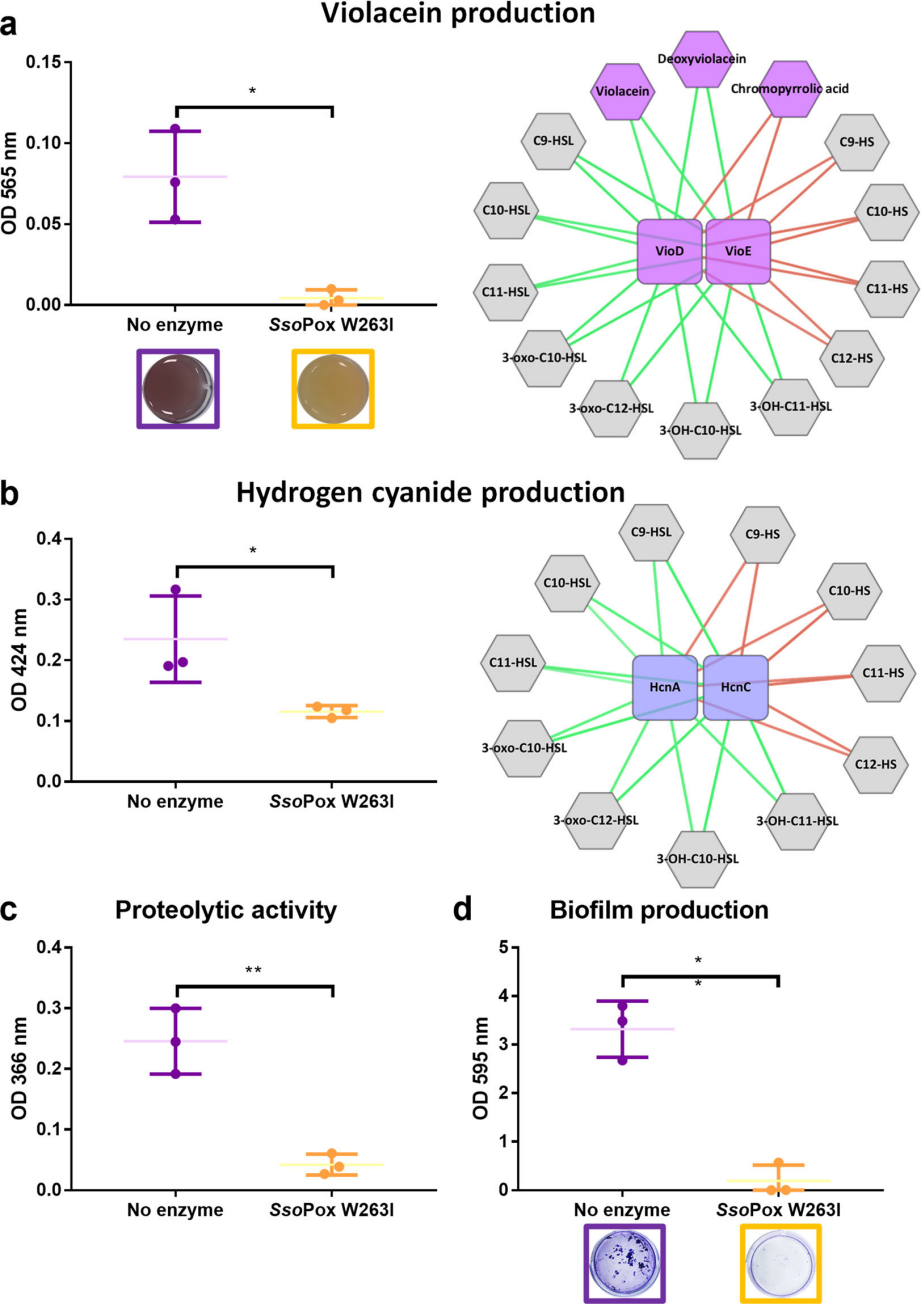
*Sso*Pox W263I inhibits QS-regulated factors production of *C. violaceum*. Mean levels of violacein (**a**) hydrogen cyanide (**b**), protease (**c**), and biofilm (**d**) production of *C. violaceum* untreated (purple) and treated cultures with 0.5 mg ml^−1^ *Sso*Pox W263I (yellow). Error bars represent the standard deviations of *n* = 3 biological replicates (each represented by one point). *P values <0.05; **P values <0.01 according to Student’s *t* test. Representative pictures of untreated (yellow squares) and lactonase treated cultures (yellow squares) (**a**) and their biofilm formation after coloration with crystal violet (**d**) are given. Network represents correlation multi-omics networks where proteins (squares) and metabolites (hexagons) involved in violacein (**a**) and hydrogen cyanide production (**b**) production and significantly impacted by *Sso*Pox W263I are positively (green) or negatively (red) connected.

To evaluate how molecular modifications could be related to phenotypic variations, the impact of *Sso*Pox W263I was investigated on QS-related behaviors of CV 12472 (Fig. 5).

**Fig. 6:**
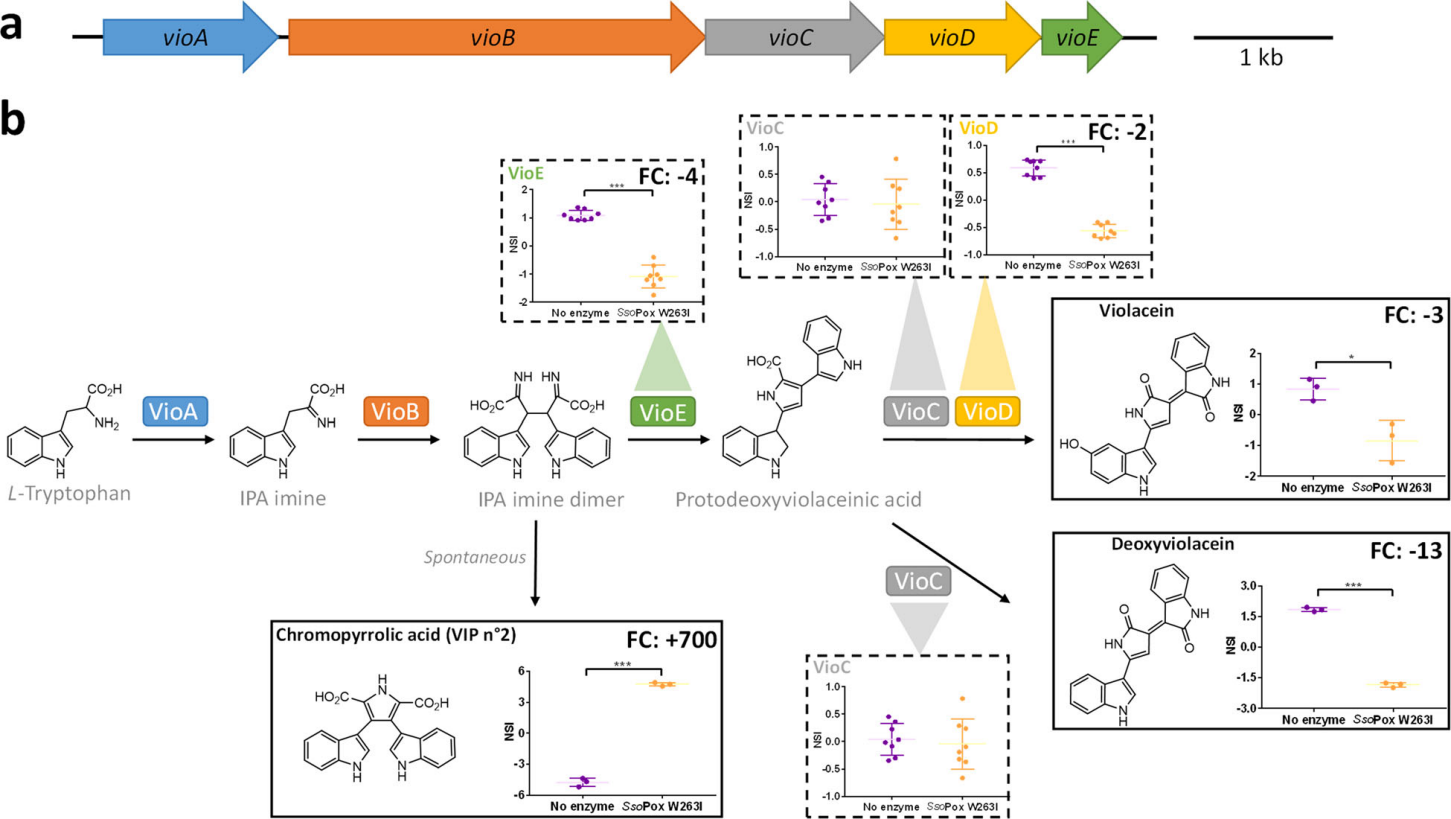
Impact of *Sso*Pox W263I on violacein biosynthesis in *C. violaceum*. **a** Organization of the violacein biosynthesis operon. Arrows indicate the direction of transcription. **b** Simplified representation of *Sso*Pox W263I impact on violacein biosynthesis by CV 12472 untreated (purple) and treated cultures (yellow). Proteins (dashed line boxes) and metabolites (full-line boxes) normalized scaled intensities (NSI) are represented as the mean and standard deviations (Metabolomic: *n* = 3 biological replicates, Proteomic: *n* = 8, 4 biological replicates × 2 technical replicates). *P value <0.05, **P value <0.01, ***P value <0.001 according to Student’s *t* test. FC fold change.

*C. violaceum* is known to produce the characteristic purple pigment violacein under the regulation of QS^19,20^. As expected, the addition of a lactonase to the culture medium suppresses the production of this pigment (Fig. 5a). Linking metabolomic and proteomic analyses allowed us to observe the impact of enzymatic treatment on different steps of the biosynthetic pathway of violacein (Fig. 6 and Supplementary Fig. 2). The main benefits of the QQ treatment were a fourfold decrease in VioE production and a 700-fold increase of chromopyrrolic acid. This latter is known to be formed spontaneously in the absence of VioE (Fig. 6)^32,33^ and was thus found to be significantly negatively correlated to this protein (Fig. 5a). Together with VioE, the abundance of VioD was significantly reduced, leading to a drastic decrease in the production of violacein and its derivatives (i.e., deoxyviolacein, proviolacein, prodeoxyviolacein, and deoxyviolacein) (Fig. 6 and Supplementary Fig. 2). Proteomic and metabolomic data matrices were further processed by regularized Canonical Correlation Analysis (rCCA)^34–36^ with a cut-off value of 0.5 chosen to create a multi-omics correlation network. This network showed significant positive relationships between the proteins VioD and VioE, on the one hand, and violacein, deoxyviolacein, and the AHLs, on the other, while these two proteins were negatively correlated with chromopyrrolic acid and the hydrolyzed AHLs (Fig. 5a).

*Chromobacterium* species are also known for their ability to produce the toxic metabolite hydrogen cyanide^37,38^. This production is under QS regulation in *C. violaceum*^21^. In this study, enzymatic treatment reduced by >50% the level of hydrogen cyanide in the supernatant (Fig. 5b). HcnC and HcnA, two proteins involved in hydrogen cyanide biosynthesis^39^, were found significantly downregulated by *Sso*Pox W263I treatment (fold change of -7.27 and -1.65, respectively, as compared to control) (Fig. 2c) and their variations were positively correlated with AHLs and negatively correlated with hydrolyzed AHLs (Fig. 5b).

Finally, a strong inhibitory effect of the lactonase on the production of proteases as well as on the biofilm formation was also observed with fold changes of -6 and -17, respectively. Nevertheless, neither proteins nor metabolites could be clearly associated with these phenotypes (Fig. 5c, d). Altogether, these results suggested that *Sso*Pox W263I constitutes a potent QQ agent for the bacterial strain CV 12472, inducing major phenotypic and metabolic changes.

### Combined omics analysis identifies anisomycin as a new QS-regulated factor

**Fig. 7:**
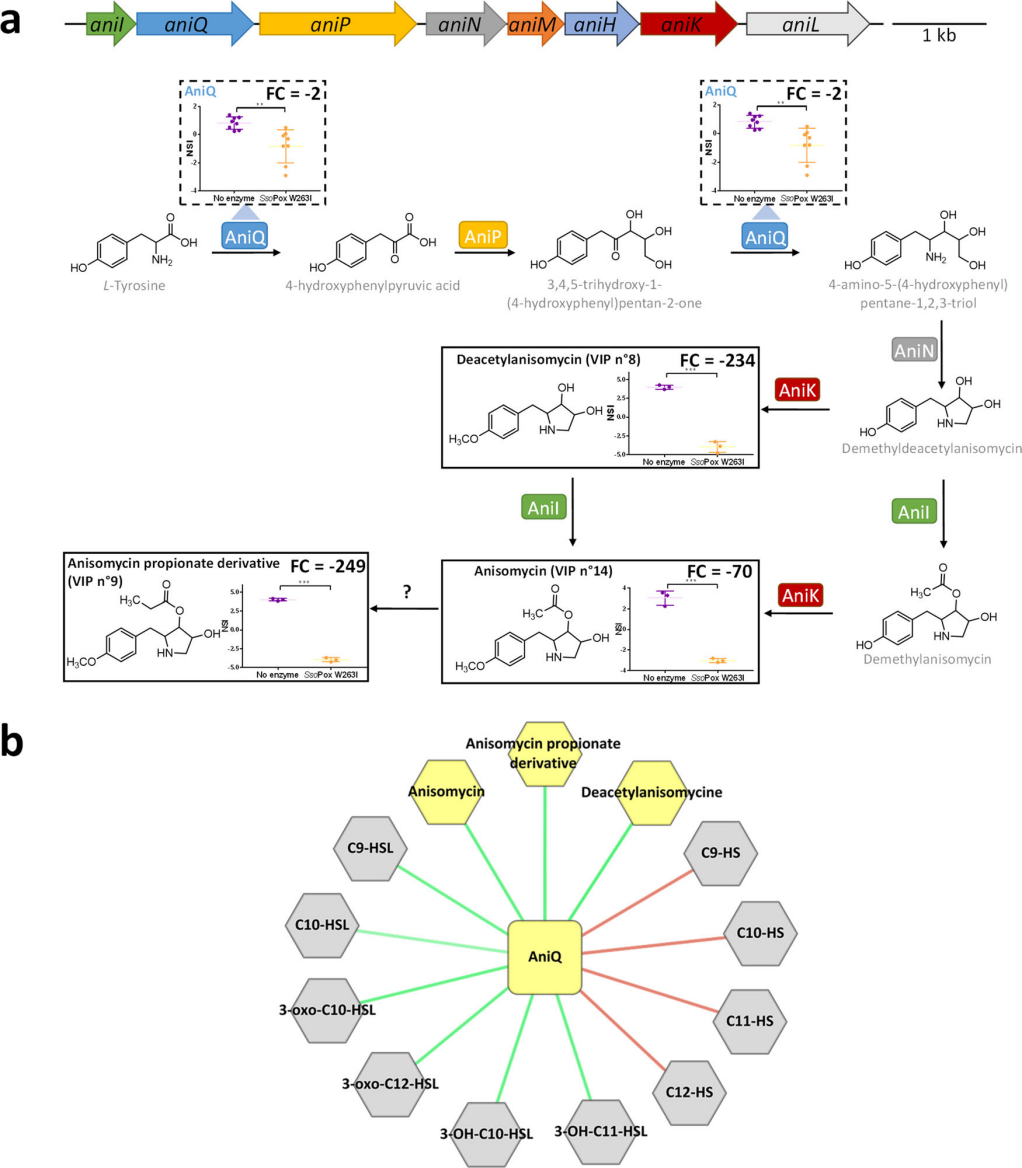
Impact of *Sso*Pox W263I on anisomycin biosynthesis in *C. violaceum*. **a** Organization of the anisomycin biosynthesis operon and impact of *Sso*Pox W263I on the anisomycin biosynthetic pathway. In operon representation, arrows indicate the direction of transcription. Pathway and nomenclature are from Zheng et al.^41^. Proteins (dashed line boxes) and metabolites (full-line boxes) normalized scaled intensities (NSI) are represented as the mean and standard deviations (metabolomic: *n* = 3 biological replicates, proteomic: *n* = 8, 4 biological replicates × 2 technical replicates). *P value <0.05, **P value <0.01, ***P value <0.001 according to Student’s *t* test. FC fold change. **b** Correlation multi-omics network of the protein AniQ (square) and metabolites (hexagons) involved in the production of anisomycin. Metabolites significantly impacted by *Sso*Pox W263I are positively (green) or negatively (red) connected with AniQ.

Besides known QS-regulated metabolites of CV 12472, further analysis of the metabolome of CV 12472 led to the identification of anisomycin, deacetylanisomycin, and anisomycin propionate derivative molecules (Figs. 3 and 7). Anisomycin is an antibiotic interfering with proteins and DNA synthesis in eukaryotes and is mainly described in *Streptomyces* species^40^. Recently, the biosynthetic gene cluster of anisomycin in *Streptomyces hygrospinosus* var. *beijingensis* has been reported. It mainly consists in five proteins (AniQ, AniP, AniN, AniI, and AniK) for the biosynthesis of anisomycin and two additional proteins being involved in its glycosylation. Homologs of AniQ, AniP, and AniN have been previously identified in *C. violaceum* genome but the production of anisomycin by *C. violaceum* and the functionality of the identified homologs have not been confirmed^41^. Here, we provided the first evidence of anisomycin production in CV 12472 and a thorough comparison of *S. hygrospinosus* ACCC40033 and CV 12472 anisomycin gene clusters. Our data confirmed the identification of the biosynthetic gene cluster of anisomycin in the CV 12472 genome between the loci CV_2255 and CV_2262. Anisomycin was also identified in cell-free supernatants (Supplementary Fig. 1b), suggesting that CV 12472 is also equipped for the secretion of this antibiotic. This analysis revealed that *C. violaceum* possesses all the required proteins to biosynthesize anisomycin from l-tyrosine although glycosylation proteins were not identified (Supplementary Fig. 3). Here, we further showed, by combining proteomic and metabolomic analyses, that anisomycin was drastically downregulated by QQ treatment (Fig. 7). Not all molecules involved in anisomycin biosynthesis were detected by the multi-omics approach, but insightful observations highlighted the impact of *Sso*Pox W263I on its production. The protein CV_2256, now identified as AniQ and involved in the formation of several biosynthetic intermediates of anisomycin, was found significantly downregulated by the enzymatic treatment (Fig. 7a). Deacetylanisomycin, a precursor of anisomycin was also downregulated, along with the anisomycin propionate derivative (also known as 3097-B1, formed by an unknown mechanism and already detected in *Streptomyces* strain SA3097^42^) (Fig. 7a). AniQ, deacetylanisomycin, anisomycin and its propionate derivative, were all positively correlated with the production of AHLs and negatively correlated with the occurrence of hydrolyzed AHLs (Fig. 7b).

Altogether, the results of this multi-omics approach showed that CV 12472 can biosynthesize anisomycin and that this production is under QS regulation.

### *C. violaceum* toxicity toward Gram-positive *Bacillus cereus* is inhibited by QQ

**Fig. 8:**
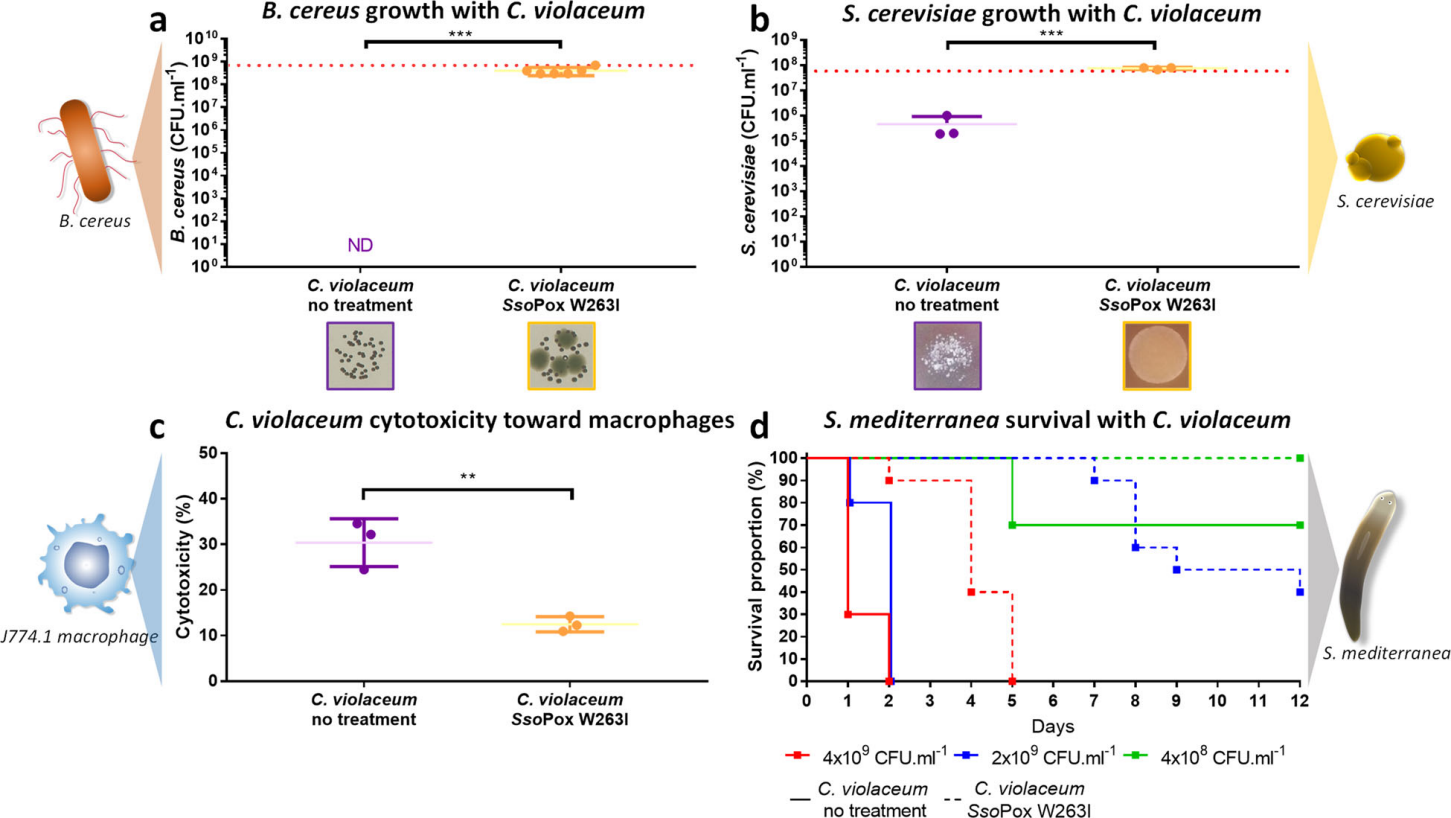
*Sso*Pox alters *C. violaceum* interaction with prokaryotic and eukaryotic organisms. **a** Mean bacterial concentration in CFU ml^−1^ of *B. cereus* after 8 h of cocultures with *C. violaceum* treated with 0.5 mg ml^−1^ *Sso*Pox W263I (yellow) and untreated cultures as negative controls (purple). Error bars represent the standard deviations of *n* = 6 experiments (three biological replicates × two technical replicates). The dotted red line represents the mean bacterial concentration of *B. cereus* monoculture. Representative pictures of dilution on LB-Agar plate (dilution factor at 10^−6^) of *B. cereus* cultures with treated cultures of *C. violaceum* with 0.5 mg ml^−1^ *Sso*Pox W263I (yellow square) and untreated cultures as negative controls (purple square) are given. ***P values <0.001 according to the Student’s *t* test. ND not detected. **b** Mean yeast concentration in CFU ml^−1^ of *S. cerevisiae* after 24 h growth on YPD-Agar infected by *C. violaceum* treated with 0.5 mg ml^−1^ *Sso*Pox W263I (yellow) and untreated as negative controls (purple). Error bars represent the standard deviations of *n* = 3 biological replicates. The dotted red line represents the mean yeast concentration of *S. cerevisiae* on non-infected YPD-Agar. Representative pictures of dilution on YPD-Agar plate (dilution factor at 10^−2^) of *S. cerevisiae* growth on the plate infected by treated cultures of *C. violaceum* with 0.5 mg ml^−1^ *Sso*Pox W263I (yellow square) and untreated cultures as negative controls (purple square) are given. ***P values <0.001 according to Student’s *t* test. ND not detected. **c** Cytotoxicity of *C. violaceum* toward J774.1 macrophages with 0.5 mg ml^−1^ *Sso*Pox W263I (yellow) or untreated as negative controls (purple) during a 1.5 h infection at a MOI of 1. Error bars represent the standard deviations of *n* = 3 replicated experiments. **P values <0.01 according to Student’s *t* test. **d** Planarian survival in the presence of *C. violaceum* treated with 0.5 mg ml^−1^ *Sso*Pox W263I (dotted line) and untreated cultures as negative controls (full line) at 4 × 10^9^ CFU ml^−1^ (red), 2 × 10^9^ CFU ml^−1^ (blue), 4 × 10^8^ CFU ml^−1^ (green). Curves represent survival proportions of ten planarians. According to log-rank (Mantel–Cox) test comparing survival curves in treated and untreated conditions *P* values <0.0001 for 4 × 10^9^ CFU ml^−1^ and 2 × 10^9^ CFU ml^−1^ conditions and *P* value = 0.0671 for 4 × 10^8^ CFU ml^−1^ concentration.

*C. violaceum* uses QS to induce antimicrobial metabolites production, including the Gram-positive targeting violacein and hydrogen cyanide^43,44^. To evaluate how QQ may affect the capacity of CV 12472 to compete with invaders, a co-culture model with *Bacillus cereus* was developed and tested with and without lactonase. Without enzymatic treatment, CV 12472 was lethal to *B. cereus* and completely inhibited its growth. Conversely, when CV 12472 was enzymatically quenched, *B. cereus* was able to grow (Fig. 8a). Similar results were obtained when *B. cereus* was grown in the presence of 50% CV 12472 supernatant, demonstrating that *C. violaceum* produced one or several extracellular toxic compounds for *B. cereus* under QS regulation (Supplementary Fig. 4a). These effects could be due to the impact of *Sso*Pox W263I on different QS-regulated mechanisms of CV 12472, such as violacein or hydrogen cyanide production. Violacein is known to disrupt Gram-positive strains’ cellular membrane integrity^45^ and hydrogen cyanide is also known to be toxic to some bacterial strains and to be involved in bacterial competition^43^. The downregulation of hydrogen cyanide and violacein production by *Sso*Pox W263I could indeed play a significant role in altering the ability of CV 12472 to eliminate *B. cereus*.

To determine whether these metabolites were involved in the toxicity against *B. cereus*, hydrogen cyanide and violacein were evaluated independently (Supplementary Fig. 4b, c). First, the amounts of violacein and hydrogen cyanide were estimated in CV 12472 supernatants: violacein was found at a concentration of 4 ± 1 µg ml^−1^ in untreated cultures and 0.3 ± 0.1 µg ml^−1^ in treated ones while hydrogen cyanide concentration was 7 ± 1 µg ml^−1^ in untreated cultures and 3 ± 1 µg ml^−1^ in treated ones. The effect of both molecules on *B. cereus* growth was assayed at the concentration found in CV 12472 supernatants. At 4 µg ml^−1^, violacein inhibited *B. cereus* growth in the same proportion as in untreated supernatants. Consistently, as for the treated cultures of CV 12472, a concentration of 0.3 µg ml^−1^ violacein did not affect the growth of *B. cereus* (Supplementary Fig. 4b). Conversely, hydrogen cyanide had no impact on *B. cereus* growth at any tested concentrations (Supplementary Fig. 4c). These results underlined that the toxic effect of CV 12472 supernatants on *B. cereus* could be mainly attributed to violacein and this effect is drastically decreased by *Sso*Pox W263I.

### *Sso*Pox inhibits *C. violaceum* toxicity toward unicellular eukaryote *Saccharomyces cerevisiae*

A competition model was developed using the yeast *S. cerevisiae* to evaluate the potential impact of QS on the interactions of CV 12472 with unicellular eukaryotic organisms (Fig. 8b). The experiment revealed that yeast growth was decreased on YPD-Agar inoculated with CV 12472. Treatment of CV 12472 with lactonase inhibited the toxicity of the bacterium toward the yeast and growth increased by a 2.2 log_10_-fold change to reach a similar level as growth on uninoculated YPD-Agar. These results showed that QS regulates the antifungal effect of CV 12472 and can be affected by AHL-targeting QQ. Different antifungal mechanisms could be involved such as violacein^46^ and cyanide production^47^; both under QS regulation in CV 12472 and inhibited by *Sso*Pox W263I. Anisomycin is also an antifungal metabolite, with known toxic activity against *S. cerevisiae*^41^. Its effect on yeast was tested to evaluate whether it could be responsible for the inhibition of growth caused by CV 12472. Interestingly, we observed that anisomycin induced the formation of small-sized colonies of *S. cerevisiae* while the number of colonies was not affected (Supplementary Fig. 5). Such a morphological change was also observed in our *C. violaceum* vs *S. cerevisiae* competition assay. Small-colony variants have been previously reported in *S. cerevisiae* treated with anisomycin^48^. Therefore, the small-colony variants observed in *S. cerevisiae* in presence of CV 12472 might be imputable to anisomycin production, but cannot explain the yeast growth defect indicating that, besides anisomycin, at least another toxic compound might be involved in the competition between CV 12472 and *S. cerevisiae*. Interestingly, cell-free supernatants obtained from treated and untreated cultures of CV 12472 induced the formation of yeast small colonies but did not alter yeast growth, suggesting that growth inhibition might require direct contact between these microorganisms (Supplementary Fig. 5).

### *Sso*Pox decreases *C. violaceum* toxicity in higher eukaryotic models: murine macrophages and planarians

*C. violaceum* is broadly found in water and soil microbiota in subtropical and tropical regions and has been recently identified as an environmental opportunistic pathogen infecting humans and animals^49^. This bacterium is thus known to be associated in rare, but frequently fatal, human infections underlining its high virulence under normal human body temperature^50,51^. To further probe the impact of *Sso*Pox W263I on the virulence of CV 12472, a cytotoxicity model using macrophages was first developed (Fig. 8c). The bacterial cytotoxicity at a multiplicity of infections (MOI) of 1, meaning a proportion of one bacterium for one macrophage, was assayed in J774.1 cells. Moderate cytotoxicity (30%) was observed in this condition, which was used to study the enzymatic effect. Interestingly, upon enzymatic exposure, the cytotoxicity decreased down to 12%, therefore suggesting a possible link between QS regulation and the observed cytotoxic effect. A similar inhibitory effect induced on the cytotoxicity of CV 12472 has previously been reported with guava leaf extract as the QS inhibitor in an infection model of HepG2 human liver carcinoma cells^52^. No cytotoxic effect was detected with supernatants of untreated or enzymatically treated CV 12472 cultures. These results indicated that cytotoxicity seemed independent from extracellular secretions, but rather implied some intracellular molecules or cellular mechanisms. We observed that *Sso*Pox W263I reduced the production of CV_1744 protein (CV-IIL) (Fig. 2c), a fucophilic lectin, which resembles PA-IIL, a QS-regulated fucose-binding lectin of *P. aeruginosa*, known to be involved in bacterial adherence to host-cell in the initial steps of infections^53,54^. This lectin could then be involved in the reduction of the cytotoxicity of CV 12472. As AHLs, and more particularly 3-oxo-C12-HSL, harbor an important toxic activity toward eukaryotic cells^55^, the degradation of these molecules induced by *Sso*Pox W263I could also play a role in the reduction of the cytotoxic effect induced by CV 12472. The decrease of CV 12472 toxicity towards macrophages upon lactonase treatment suggested that QQ is a promising alternative approach to tackle human and animal infections by *C. violaceum*.

In *C. violaceum*, QS regulates the biosynthesis of toxic metabolites, including violacein, hydrogen cyanide, and anisomycin. To assess whether QS may contribute to *C. violaceum* competition with animals, we used an aquatic model organism, the freshwater flatworm *Schmidtea mediterranea*. In recent decades, planarians have emerged as relevant invertebrate models for toxicology and ecotoxicology studies and have been used to evaluate neurodegenerative diseases as well as the toxicity of environmental pollutants and neurotoxic chemicals^56–59^. Moreover, planarians have been previously described as particularly resistant to bacterial infections by eliminating bacteria mainly through phagocytosis^60^. Here, we used lifespan assay to evaluate the toxicity of *C. violaceum* on *S. mediterranea*. Bacteria were first grown with or without lactonase and then incubated with planarians. Planarians were not able to eliminate the bacterial threat in either of these conditions as the bacterial concentrations remained equivalent to those of the worm-free control for 12 days (Supplementary Fig. 6). We showed that *C. violaceum* rapidly kills planarians from a bacterial load of 4 × 10^9^ CFU ml^−1^ (Fig. 8d). Interestingly, lactonase-mediated quenching decreased bacterial toxicity and significantly enhanced worms’ survival, leading to complete inhibition of mortality at 4 × 10^8^ CFU ml^−1^.

These results underscored that QS can provide *C. violaceum* important advantages for environmental interactions, such as better fitness when competing against animals as planarians. In previous studies, violacein has been described for its nematicide effect against *Caenorhabditis elegans*^61,62^ and anisomycin, a translation inhibitor in eukaryotes has been shown to affect wound healing in planarians^63^ but other factors might also be involved in toxicity towards planarians. Consistently, we showed that QS mediates the toxicity of *C. violaceum* in *S. mediterranea* and that enzymatic quenching can reduce the virulence of this bacterial strain toward planarians. The toxic mechanisms produced by CV 12472 and killing planarians remain unknown but they probably differ from other toxic models that require bacterial ingestion since the bacterial load remains unchanged along the experiment and that cell-free supernatants also showed toxicity (Supplementary Fig. 7).

## Discussion

QS disruption driven by lactonase *Sso*Pox W263I was previously shown to modulate microbial communities from soil and lake water samples thus affecting ecological behaviors including biofilm formation, colonization or biocorrosion. To further address the direct and indirect impacts of this enzyme on diverse bacteria able or not to produce or sense AHLs, we evaluated the molecular and phenotypic mechanisms involved in social interactions of CV 12472, a proteobacterium representative from natural environments.

The production of the main AHLs of CV 12472, namely C10-HSL and 3-OH-C10-HSL was first confirmed, along with C9-HSL, C11-HSL, 3-OH-C11-HSL, 3-oxo-C10-HSL, 3-oxo-C12-HSL which had never been described before in this strain. All AHLs were present both in whole-cell extracts and cell-free supernatants. *Sso*Pox W263I quenching effect was confirmed by the diminution of the production of these AHLs in treated samples and the detection of the hydrolyzed forms of some of them (C9-HS, C10-HS, C11-HS, and C12-HS, respectively).

Common QS-regulated traits, such as exoprotease production and biofilm formation, were shown to be inhibited by the enzymatic treatment. Moreover, *Sso*Pox W263I also induced a decrease in the production of both hydrogen cyanide and violacein observed at proteomic, metabolomic, and phenotypic levels.

The biosynthesis of anisomycin was unexpectedly detected in CV 12472 (in whole-cell and cell-free supernatant extracts) and the presence of the corresponding biosynthetic gene cluster (only previously hypothesized) was confirmed. A downregulated impact of the QQ treatment was observed for one protein involved in anisomycin biosynthesis, as well as the decrease of the final metabolites of this biosynthetic pathway, showing that anisomycin production is under QS regulation in this strain.

Considering the broad impact of QS on the synthesis of toxic metabolites as well as the impact of enzymatic QQ (Supplementary Fig. 8), the effect of the lactonase *Sso*Pox W263I on *C. violaceum* social interactions was evaluated in prokaryotic and eukaryotic competition assays. Treatment of CV 12472 by the lactonase was found to drastically inhibit toxicity toward a Gram-positive bacterium, yeast, and higher eukaryotic organisms using in vitro and in vivo models. Competition models with *B. cereus* and *S. cerevisiae* revealed that treatment with the QQ agent in *C. violaceum* decreased the production of extracellular antimicrobial compounds. We found that CV 12472 toxic effect on *B. cereus* was mainly ascribable to violacein and that anisomycin affected the morphology of *S. cerevisiae*, while its sole action did not alter yeast growth.

Besides its impact on interactions with bacteria and yeast, lactonase treatment was found to decrease CV 12472 cytotoxicity toward J774.1 murine macrophages and reduce mortality in planarians. These investigations confirmed the toxicity of CV 12472 in higher eukaryotic models and suggested that QS may be a relevant target to decrease *C. violaceum* toxicity towards higher eukaryotes.

This study draws a complete overview of the ability of lactonase-mediated QQ to disrupt bacterial competition toward other organisms. Under our experimental conditions, the effects of *Sso*Pox W263I observed on CV 12472 cultures provide essential insights into the role of QS in interkingdom relations and pave the way for further investigations, particularly concerning the role of QQE on bacterial populations. Here, the results obtained by combining molecular analyses and simplistic competitions assays showed evidence that lactonase-mediated QS disruption may affect bacteria in both inter- and intra-kingdom interactions. Complex environments and interactions are hard to reproduce under laboratory conditions due to challenges in combining different organisms' growth requirements. To fully understand the role of QS in multispecies communities and dynamics, metagenomic data would need to be examined in light of metabolomic analyses to identify the molecular causes of microscopic changes and population dynamics.

## Methods

### Microbial strains and growth conditions

Experiments were conducted using *Chromobacterium violaceum* ATCC 12472, *Bacillus cereus* ATCC 1457, and *Saccharomyces cerevisiae* ATCC 9763. Bacteria were grown in Luria Bertani (LB) medium (10 g l^−1^ NaCl, 10 g l^−1^ tryptone, 5 g l^−1^ yeast extract) at 37 °C and yeast were grown in Yeast extract–Peptone–Dextrose (YPD) medium (10 g l^−1^ yeast extract, 20 g l^−1^ peptone, 10 g l^−1^ glucose) at 30 °C. For *C. violaceum*, bacteria were inoculated from a single colony and pre-cultivated for 6 h at 37 °C in LB medium with agitation at 350 rpm. Then, precultures were diluted by a 1 000 factor in LB medium in 12-well plates, and cultures were incubated at 37 °C under agitation at 350 rpm for 16 h. The enzyme was added, when indicated, at a final concentration of 0.5 mg ml^−1^.

### Protein production and purification

*Sso*Pox W263I variant was used as QQ enzyme and productions were performed as follow^18,64,65^. Briefly, cells of *Escherichia coli* Bl21 (DE3)-pGro7/GroEL (TaKaRa Bio, Shiga, Japan), carrying plasmid pET22b-*Sso*Pox-W263I, were cultivated in ZYP-5052 medium complemented with 100 µg ml^−1^ ampicillin and 34 µg ml^−1^ chloramphenicol at 37 °C to a final OD 600 nm of 0.8–1. l-arabinose was added to induce the expression of chaperone proteins at a final concentration of 0.2% (w/v). Simultaneously, 0.2 mM of CoCl_2_ was added, and the temperature was reduced down to 23 °C. Cells were harvested by centrifugation (4400 × *g*, 4 °C, 20 min) after 20 h of incubation. The pellet was then resuspended in lysis buffer (50 mM HEPES pH 8, 150 mM NaCl, 0.25 mg ml^−1^ lysozyme, 0.1 mM phenylmethylsulfonyl fluoride (PMSF), and 10 µg ml^−1^ DNAseI) and stored at −80 °C for 16 h. Cells were thawed for 15 min at 37 °C and lysed by three steps of 30 s sonication (QSonica sonicator Q700, amplitude at 45). Centrifugation was used to remove cell debris (21,000 × *g*, 4 °C, 20 min). The resulting extract was incubated for 30 min at 80 °C and centrifuged to remove the precipitate (21,000 × *g*, 4 °C, 30 min). The enzyme was concentrated by overnight incubation at 4 °C in 75% ammonium sulfate. After resuspension in activity buffer (50 mM HEPES pH 8, 150 mM NaCl), ammonium sulfate was eliminated by desalting step (HiPrep 26/10 desalting, GE Healthcare, ÄKTA Avant, Chicago, IL, USA). The protein sample was concentrated using 30 kDa centricon, and then purified to homogeneity using a size-exclusion chromatography column (Hiload 16/600 Superdex™ 75 pg, GE Healthcare, ÄKTA Avant). Protein purity was checked by migration on 10% SDS-PAGE, and protein concentration was measured using a spectrophotometer NanoDrop 2000 (Thermo Fisher Scientific, Waltham, MA, USA).

### Proteome extraction

After 16 h of culture in LB with or without enzyme, bacteria were collected. The samples were centrifuged 5 min at 11,000 × *g*, the supernatant was discarded, and the pellet was washed twice in phosphate-buffered saline solution (PBS). To prepare total protein samples, the supernatant was discarded and the pellet was resuspended in UTS buffer (8 M urea, 2 M thiourea, 100 mM NaCl, 25 mM Tris, pH 8.0, and protease inhibitors (Roche, Pleasanton, CA, USA, Complete, EDTA free)). The samples were sonicated three times for 1 min (QSonica Q700, amplitude at 45 with 30 s intermittence) and were then centrifuged at 13,000 × *g* for 30 min at room temperature. In a second time, the supernatant was transferred in dialysis cassettes (Slide-A-lyser Dialysis Cassettes 2 K MWCO, Thermo Fisher Scientific) and placed in 2 l of Urea/Ambic buffer (1 M urea, 50 mM ammonium bicarbonate) for 4 h followed by one night in 2 l of fresh Urea/Ambic buffer. The protein concentration in each sample was then determined using Bradford’s assay. Samples containing 50 µg of proteins were prepared to a final volume of 45 µl using Urea/Ambic buffer. For disulfide bond reduction, 1 µl of DTT (0.5 M dithiothreitol in Urea/Ambic buffer) was added and the samples were incubated for 1 h at 30 °C. For alkylation, 2 µl of IAA (0.5 M iodoacetamide in Urea/Ambic buffer) was then added before a 1 h incubation in the dark. Proteins were then digested by adding 2 µl of trypsin for a ratio 1:25 (w:w), and the samples were incubated overnight at 37 °C. Finally, to remove detergents and clean the samples, Pierce detergent removal spin column (PierceTM, Thermo Fisher Scientific) and C18 spin column (PierceTM) were used according to the manufacturer’s recommendations. The result of this preparation was suspended in acetonitrile 70%. The solutions were evaporated and resolubilized in 50 µl of eluent (0.1% formic acid) to have 1 µg/1 µl solutions of peptides.

### Label-free quantitative nano-LC-MS/MS proteomic analysis

Protein digests were first separated by ultraperformance liquid chromatography (UPLC) using the NanoAcquity UPLC System (Waters, Elstree, UK) connected to a Synapt G2Si Q-TOF ion mobility hybrid mass spectrometer (Waters). The chromatographic system was used in 1D configuration with an analytical column (ACQUITY UPLC M-Class peptide CSH C18 Column, 130 Å 1.7 µm, 75 µm × 100 mm, Waters) after a trapping column (ACQUITY UPLC M-Class Symmetry C18 Trap Column, 100 Å 5 µm 2 G V/M, 180 µm × 20 mm, Waters). Eluted peptides were then separated using a 100 min gradient (300 nl min^−1^; 0.5 to 40 % acetonitrile–0.1% formic acid). Data-independent MS/MS analysis was performed with the ion mobility feature (HDMSe method). The parameters of the ion source were capillary voltage at 3 kV, sampling cone voltage at 40 V, ion source temperature at 90 °C, cone gas flow at 50 l h^−1^. Transfer collision low energy was set to 5 V, trap collision low energy was set to 4 V. The high energy ramp was applied from 4 to 5 V for the trap collision and from 19 to 45 V for the transfer collision enabling fragmentation of the ions after ion mobility cell and before the time-of-flight (TOF) MS. The On-column sample load was 800 ng. Each sample was injected in duplicate.

The files acquired were imported into Progenesis QI software v. 2.0 (Nonlinear Dynamics, Newcastle, UK) for label-free quantification analysis. The data were automatically aligned and normalized. Processing parameters were 150 counts for the low energy threshold, 30 counts for the elevated energy threshold. The database used to identify peptides contains the protein sequences of *C. violaceum* ATCC 12472 (UniProt, 08/05/2018, 4397 sequences). Search tolerance parameters were as follows: peptide and fragment tolerance, 15 ppm, FDR < 1 %; minimum ion matching requirements were three fragments per peptide, seven fragments per protein, and two peptides per protein. The enzyme specificity was trypsin allowing one missed cleavage. The accepted modifications were carbamidomethyl of cysteine (fixed), oxidation of methionine (variable), carbamyl of lysine and *N*-terminal (variable), deamidation (variable) of asparagine and glutamine. The protein normalization was performed according to the relative quantitation using non-conflicting peptides. To determine the significance of changes between samples, a significant ANOVA (*P* value < 0.05) and a fold change superior to 1.5 were used as thresholds to define differently expressed proteins.

Functional annotation of the differentially expressed proteins was carried out using DAVID annotation tool (https://david.ncifcrf.gov/), the gene ontology (GO) database in the UniProt Knowledgebase (UniProtKB) and bibliography. Of note, as *C. violaceum* is known to be highly virulent at normal human body temperature, we chose to grow the bacterium at 37 °C in all our experiments. This must be considered when comparing our “omics” results with previously reported data mainly performed at 30 °C.

### Metabolite extraction

In total, 25 ml of bacterial culture (triplicates) in LB with or without enzyme were extracted using 5 ml of ethyl acetate (LC-MS grade, Sigma-Aldrich, St. Louis, MO, USA). After 30 min of sonication, the upper organic phase was collected and dried in vacuo. The resulting dried extracts were solubilized in methanol (LC-MS grade, Sigma-Aldrich), filtered (0.2 µm), dried under a nitrogen flow, and were then kept in nitrogen at −20 °C. For more details, see ref. ^66^. For supernatant extraction, 25 ml of bacterial cultures in LB were centrifuged, the recovered supernatant was filtered (0.22 µm) and extracted using 5 ml of ethyl acetate. The upper organic phase was collected and the same procedure was applied as for whole-culture extractions.

### LC-ESI-MS and MS/MS data acquisition

Samples were prepared by solubilizing the dry extracts in 1 ml of LC-MS grade methanol. Six medium blanks were also prepared using the same protocol but without bacteria. Four quality control samples (QCs) were prepared by mixing an aliquot of all the samples. Samples and medium blanks were randomly injected, and a QC was injected every three samples. At the beginning of the injection sequence, two analytical blanks (only methanol) and a QC were injected before the samples.

Samples were analyzed by UPLC-ESI-QToF-MS in positive mode. The UPLC−MS instrumentation consisted of a Dionex Ultimate 3000 Rapid Separation (Thermo Fisher Scientific) chromatographic system coupled with a QToF Impact II mass spectrometer (Bruker Daltonics, Mannheim, Germany). The separations were carried out on a reverse-phase column (150 × 2.1 mm, 1.7 μm, Kinetex Phenyl-Hexyl; Phenomenex, Torrance, CA, USA) equipped with a pre-column (SecurityGuard cartridge, Phenomenex) at a temperature of 40 °C. The injection volume was 5 μl, and the flow rate was set at 0.5 ml min^**−1**^. The autosampler temperature was set at 4 °C, and the injection volume was 5 μl. Mobile phases were: (A) water and (B) acetonitrile (Chromasolv; Sigma-Aldrich) containing each 0.1% (v/v) of formic acid (ultra grade; Fluka, Fischer Scientific). The elution gradient started at 5% B, maintained for 2 min, then increased to 100% B (linear ramp) in 8 min, and maintained for 4 min; then back to 5% B (linear ramp) over 0.01 min and maintained 1.99 min, for a total run time of 16 min.

The capillary voltage of the MS spectrometer was set at 4500 V (positive mode), and the nebulizing parameters were set as follows: nebulizing gas (N_2_) pressure at 0.4 bar, drying gas (N_2_) flow at 4 l min^−1^, and drying temperature at 180 °C. Mass spectra were recorded from *m/z* 50 to 1200 at a mass resolving power of 25,000 full width at half-maximum (FWHM, *m/z* = 200) and a frequency of 2 Hz. Tandem mass spectrometry analyses were performed thanks to collision-induced dissociation (CID) with a collision energy of 25 eV. A solution of formate/acetate forming clusters was automatically injected before each sample for internal mass calibration, and the mass spectrometer was calibrated with the same solution at the beginning of the sequence.

### Metabolomic data processing and analysis

LC-MS raw data were converted into “.netCDF“ files using DataAnalysis v. 4.3 (Bruker, Mannheim, Germany) and processed with MZmine v. 2.53^67^. All steps and parameters used to obtain the final data matrix are listed in Supplementary Table 1. In a second time, the data were submitted to two filtering steps using an in-house script on R and final manual filtering. Filtering consisted in removing successively experimental and analytical bias according to signal/noise ratio (using blanks) and coefficient of variation (using QCs). For multivariate analyses, the resulting data matrix was log_10_-transformed, mean-centered, and analyzed using MetaboAnalyst 4.5 online^68^. Data were analyzed using Principal Component Analysis (PCA) and Partial Least Squares (PLS-DA) discriminant analysis. From the PLS-DA, the most discriminating *m/z* features (VIPs) were selected according to their VIP score. This statistical model was evaluated using a permutation test and a cross-validation analysis of variance (MetaboAnalyst 4.5 online) The comparison of metabolites of interest between the two culture conditions was carried out using a *t* test (GraphPad Prism v. 7.04).

### Feature annotation via molecular networking approach

LC-MS/MS raw data (samples and standards) were converted into “.mzXML” files using DataAnalysis. The Internet platform GNPS (http://gnps.ucsd.edu) was used to create molecular networking of related compounds based on the similarity of their MS/MS fragmentation patterns^23^. Similar chemical structures share similar fragmentation pathways in mass spectrometry leading to identical fragments and losses^69^. The parameters used were: Precursor Ion Mass Tolerance (Da): 0.02, Fragment Ion Mass Tolerance (Da): 0.02, Minimum Cosine Score: 0.62, Minimum Matched Fragment Ions: 4 and Network TopK: 10. Data were imported and treated offline (Cystoscape v 3.7.0). Two methods were used for the annotation of a specific *m/z* feature: (1) Searching the most probable molecular formula with DataAnalysis software with the “*Smartformula*” package, (2) Using online databases, such as PubChem (https://pubchem.ncbi.nlm.nih.gov), Lipidmaps (http://www.lipidmaps.org), CEU mass mediator (http://ceumass.eps.uspceu.es), Metlin (https://metlin.scripps.edu)^70^, or SIRIUS 4.4.17^71^. Finally, a careful analysis of the MS/MS fragmentation pattern in comparison with the literature data was performed to confirm the putative annotation.

### Multi-omic data processing

Proteomic and metabolomic data matrices were processed by regularized Canonical Correlation Analysis (rCCA)^34–36^ with the R package MixOmics following the protocol found on the website http://mixomics.org/. The shrinkage method was used to adjust the regularization parameters *λ*1 and *λ*2. A cut-off value of 0.5 was chosen to create a molecular network with the function *write.graph()* under the format “.graphml”. The resulting network was exported and treated using Cytoscape v. 3.7.0.

### Lactonase activity measurement

The activity was measured on C9-HSL,C10-HSL, C11-HSL, C12-HSL, and 3-OH-C10-HSL at ambient temperature using a colorimetric pH-based assay^72^. Briefly, the hydrolysis of the lactone ring leads to the acidification of the solution, which is followed by the absorbance modification of a pH indicator (cresol). For kinetic parameters, the degradation of lactones at different concentrations by each enzyme in a cresol-buffered solution (1.25 mM Bicine, 150 mM NaCl, 0.2 mM CoCl_2_, 0.25 mM cresol purple, 3.5% for C9-HSL, C11-HSL, and C12-HSL or a minimum of 1.5% DMSO for C10-HSL and 3-OH-C10-HSL, and pH 8.3) was followed in 200 μl at 577 nm using a plate reader (Synergy HT, BioTek,, Winooski, VT, USA). For C9-HSL and C11-HSL, 10 mg ml^−1^ of *Sso*Pox W263I were used, and 20 mg ml^−1^ for C10-HSL, C12-HSL, and 3-OH-C10-HSL. The activity of *Sso*Pox W263I was not assayed on 3-OH-C11-HSL due to its commercial unavailability.

### Analysis of quorum sensing-regulated factors

QS-regulated factor productions were determined in vitro after 16 h of culture at 37 °C with agitation (350 rpm) in the presence of 0.5 mg ml^−1^ *Sso*Pox W263I and without enzyme.

### Biofilm formation

Biofilm was measured using crystal violet^73^. Planktonic cells were removed, and wells were washed once with PBS. After drying the plates at 37 °C until complete evaporation of PBS, crystal violet 0.5% was added, and following a wash with PBS, the remaining colorant was dissolved using ethanol 100%. Then, 200 µl were collected and absorbance was measured at 595 nm using a microplate reader (Synergy HT, BioTek,, Winooski, VT, USA).

### Proteolytic activity

Cell-free culture supernatants were prepared by centrifugation (10 min, 12,000×*g*, room temperature). Protease activity was measured using azocasein (Sigma-Aldrich)^74^. Then 25 µl of cell-free supernatant was added to a mixed solution of 675 µl PBS solution pH 7 and 50 µl of azocasein (30 mg ml^−1^ in water). Samples were incubated for 2 h at 37 °C, the reaction was then stopped by adding 125 µl of 20% (w/v) trichloroacetic acid. The samples were centrifuged (10 min, 10,000 × *g*, room temperature), and the absorbance of 200 µl of supernatant was measured at 366 nm using a plate reader (Synergy HT, BioTek).

### Violacein production

Violacein was extracted from 500 µl of cell-free supernatant by adding 500 µl of ethyl acetate^75^, the mixture was vortexed then centrifuged (5 min, 10,000 × *g*, room temperature), and 200 µl of the upper phase was collected. The absorbance was measured at 565 nm using a microplate reader (Synergy HT, BioTek).

To estimate the concentration of violacein in the supernatant, OD_565_ values were compared to a standard curve made with pure violacein isolated from *C. violaceum* (Coger, Paris, France) resuspended in LB at a known concentration ranging from 1 to 40 µg ml^−1^.

### Hydrogen cyanide production

Extracellular cyanide production was measured by using methemoglobin^76^. The formation of cyanomethemoglobin complex induces a shift in the maximum absorbance spectrum from 407 nm (free methemoglobin) to 424 nm (cyanomethemoglobin complex). Methemoglobin reagent was prepared by dissolving 85 mg of bovine hemoglobin in 12.5 ml of NaNO_2_, then 12.5 ml of phosphate buffer 0.1 M were added to the methemoglobin reagent. In total, 50 µl of cell-free supernatants were diluted with 140 µl of Millipore water. The absorbance background of the samples was measured at 424 nm using a plate reader (Synergy HT, BioTek). Subsequently, 10 µl of methemoglobin reagent was added to give a final volume of 200 µl and let react for 30 min. Finally, the absorbance of the samples was measured at 424 nm. The absorbance background was then subtracted from the final measure.

To estimate the concentration of hydrogen cyanide in the supernatant, OD_424_ values were compared to a standard curve realized with potassium cyanide (Sigma) resuspended in LB at a known concentration ranging from 0.5 to 8 µg ml^−1^.

### Competition assay with *B. cereus*

After 16 h culture in LB with or without enzyme for *C. violaceum* and an overnight preculture (16 h) in LB at 37 °C with agitation for *B. cereus*, bacteria were inoculated at 1/100 in LB medium. The cocultures were incubated at 37 °C under agitation at 350 rpm for 8 h. Then the samples were serially diluted and plated on an LB-Agar plate. Colony-forming units (CFUs) were counted after overnight incubation at 37 °C.

### Bactericidal effect of *C. violaceum* supernatant against *B. cereus*

To evaluate the bactericidal effect of *C. violaceum* supernatant against *B. cereus*, supernatant from a 16 h culture in LB with or without enzyme of *C. violaceum* was collected by centrifugation and filtered at 0.22 µm. An overnight preculture of *B. cereus* was inoculated at 1/1000 corresponding to 3 × 10^5^ CFU ml^−1^ in LB with 50% of *C. violaceum* supernatant. After an incubation of 8 h at 37 °C with agitation at 350 rpm, the culture was serially diluted and plated on LB-Agar plates. After a night at 37 °C, CFUs were counted.

### Toxicity effect of hydrogen cyanide and violacein against *B. cereus*

To evaluate the toxic effect of hydrogen cyanide and violacein supernatant against *B. cereus*, KCN (Sigma-Aldrich) and violacein (Coger) were resuspended in LB at the required concentration. An overnight preculture of *B. cereus* was inoculated at 1/1000 (3 × 10^5^ CFU ml^−1^) in LB with 50% of KCN or violacein solution. After an incubation of 8 h at 37 °C with agitation at 350 rpm, the culture was serially diluted and plated on LB-Agar plates. After 16 h at 37 °C, CFUs were counted.

### Antifungal activity of *C. violaceum* toward *S. cerevisiae* assay

To evaluate the toxic effect of *C. violaceum* on *S. cerevisiae* growth, 1.5 ml of a 16 h culture with or without enzyme of *C. violaceum* were collected and centrifuged for 5 min at 10,000 × *g*. Supernatants were discarded and pellets were washed with PBS buffer. After a centrifugation step (10,000 × *g*, 5 min, room temperature), the pellets were resuspended in 1.5 ml of melted YPD-Agar (10 g l^−1^ yeast extract, 20 g l^−1^ peptone, 10 g l^−1^ glucose, 1.5% agar) and poured in a 12-well plate. *S. cerevisiae* was inoculated from a single colony and pre-cultivated during 16 h at 30 °C in YPD medium with agitation at 350 rpm. In total, 10 µl of these cultures and tenfold serial dilutions were spotted onto *C. violaceum* inoculated YPD-Agar. After 16 h at 30°C, CFUs were counted.

### Toxicity effect of *C. violaceum* supernatants and anisomycin against *S. cerevisiae*

To evaluate the bactericidal effect of *C. violaceum* cell-free supernatants against *S. cerevisiae*, supernatants from a 16 h culture in LB with and without enzyme of *C. violaceum* were collected by centrifugation and filtered at 0.22 µm. The supernatants were added at a final concentration of 50% (v/v) in YPD-Agar 1.5% at 56 °C and poured in a 12-well plate. In all, 10 µl of an overnight preculture of *S. cerevisiae* and tenfold serial dilutions were spotted on anisomycin-containing YPD-Agar. After 16 h growth at 30 °C, *S. cerevisiae* CFUs were counted.

### Toxicity effect of anisomycin against *S. cerevisiae*

To evaluate the toxic effect of anisomycin against *S. cerevisiae*, anisomycin from *Streptomyces griseolus* (Sigma-Aldrich) was added in a concentration ranging from 50 nM to 500 µM in YPD-Agar 1.5% at 56 °C and poured in a 12-well plate. In total, 10 µl of an overnight preculture of *S. cerevisiae* and tenfold serial dilutions were spotted on anisomycin-containing YPD-Agar. After 16 h at 30 °C, CFUs were counted.

### LDH cytotoxicity assay toward J774.1 macrophages

J774.1 macrophages (murine macrophage) were grown in 75 cm^2^ ventilated flask in Roswell Park Memorial Institute (RPMI) 1640 Medium Gibco^TM^ (Thermo Fischer Scientific, Waltham, MA, USA) supplemented with 10% fetal bovine serum (FBS) Gibco^TM^ (Thermo Fischer Scientific) and 1% penicillin/streptomycin Gibco^TM^ (Thermo Fischer Scientific) at 37 °C in a humidified atmosphere containing 5% CO_2_. Cells were transferred to a 50-ml tube and centrifuged at 750 × *g* for 5 min at 4 °C. In a second time, cells were resuspended in RPMI with 10% FBS and centrifuged again to wash out any trace of antibiotics. Cells were seeded in a 96-well plate at 10^5^ cells per well in 100 µl of RPMI with 10% FBS. Plates were incubated for 20 h at 37 °C with 5% CO_2_. Cells of *C. violaceum* following 16 h of culture with and without enzyme were diluted in RPMI with 10% FBS and added to J774.1 cells; the different multiplicity of infection (MOI) of 1, 10, and 100 were tested and MOI of 1 was selected as MOI of 10 and 100 led to cytotoxic levels too high to be evaluated properly. In order to assess supernatant toxicity, 10 µl of supernatant of the bacterial culture was added to 10^5^ J774.1 cells. After a centrifugation step (10 min, 500 × *g*, 4 °C) to bring bacteria in contact with macrophages, the plate was incubated for 1.5 h at 37°C with 5% CO_2_. Supernatants were collected after centrifugation of (5 min, 200 × *g*, 4 °C). Cytotoxicity induced in J774.1 cells was quantitated by measuring the release of the cytosolic enzyme lactate dehydrogenase (LDH) in the culture medium using CyQUANT™ LDH Cytotoxicity Assay Kit (Thermo Fischer Scientific) according to the manufacturer recommendations. Maximum LDH release was measured with the lysis buffer provided by the kit, added 45 min before the end of the infection. Negative control was measured by adding PBS to non-infected macrophage cells.

### Toxicity assay using planarians

Freshwater planarians belonging to the *Schmidtea mediterranea* species (asexual clonal line ClW4) were used for the experiment. The planarians were maintained in autoclaved water at 19 °C in the dark and fed twice a week with calf liver. The animals were starved for at least one week prior to the experiments. The water was changed every 2 days and did not contain antibiotics. Worms were manually selected to fall within a certain range of size, around 0.8–1 cm in length. Cells of *C. violaceum* from a 16-h culture with and without enzyme were washed twice in PBS and then resuspended at the desired concentrations in autoclaved water. In all, 4 ml of the bacterial solution was transferred into a 12-well plate. Five planarians were added in each well and the plate was incubated at 19 °C in the dark. Survival was monitored over 12 days. Experiments were conducted in duplicate. To follow the evolution of bacterial concentration in the wells, samples of tap water were taken from worm-containing wells and control wells without worms over time. Then, the samples were serially diluted and plated on an LB-Agar plate. Colony-forming units (CFUs) were counted after 48 h incubation at room temperature.

### Toxicity effect of *C. violaceum* supernatants against *S. mediterranea*

To evaluate the bactericidal effect of *C. violaceum* supernatants against *S. mediterranea* planarian, supernatants from a 16-h culture in LB with and without enzyme of *C. violaceum* were collected by centrifugation and filtered at 0.22 µm. Freshwater planarians belonging to the *Schmidtea mediterranea* species (asexual clonal line ClW4) were used for the experiment. The planarians were maintained in autoclaved water at 19 °C in the dark and fed twice a week with calf liver. The animals were starved for at least one week prior to the experiments. The water was changed every 2 days and did not contain antibiotics. Worms were manually selected to fall within a certain range of size, around 0.8–1 cm in length. Supernatants of *C. violaceum* from a 16 h culture with and without enzyme were resuspended at the desired concentrations in autoclaved water. In total, 4 ml of the supernatant solution was transferred into a 12-well plate. Five planarians were added in each well, and the plate was incubated at 19 °C in the dark. Survival was monitored over 12 days. Experiments were conducted in duplicate.

### Reporting summary

Further information on research design is available in the Nature Research Reporting Summary linked to this article.

## Supplementary information

Supplementary Information

Supplementary Data 1

Reporting Summary

## Data Availability

All data used in this study, including raw data, are available upon request to the corresponding authors.

## Code availability

Codes used in this study are available upon request to the corresponding authors.
