# Supplementary material for: Disrupting quorum sensing alters social interactions in Chromobacterium violaceum
Source: NPJ Biofilms Microbiomes. 2021 Apr 22;7:40. doi: 10.1038/s41522-021-00211-w (PMC8062528; doi:10.1038/s41522-021-00211-w)
Supplement: Supplementary file 1 — Supplementary Information [file 41522_2021_211_MOESM1_ESM.pdf]

Supplementary information

Title: Disrupting quorum sensing alters social interactions in *Chromobacterium violaceum*

Authors: Sonia Mion[1°], Nathan Carriot[3°], Julien Lopez[3], Laure Plener[2], Annick Ortalo-Magné[3], Eric Chabrière[1*], Gérald Culioli[3,4*], David Daudé[2*]

[1]Aix Marseille University, Institut de Recherche pour le Développement, Assistance Publique - Hôpitaux de Marseille, Microbes Evolution Phylogeny and Infections, Institut Hospitalo-Universitaire-Méditerranée Infection, Marseille, France

[2]Gene&GreenTK, Marseille, France

[3]Université de Toulon, MAPIEM, EA 4323, Toulon, France

[4]Institut Méditerranéen de Biodiversité et d'Ecologie marine et continentale, UMR CNRS-IRD, Avignon Université, Aix-Marseille Université, Avignon, France

°These authors contributed equally to this work

***Corresponding authors:** david.daude@gene-greentk.com; gerald.culioli@univ-tln.fr; eric.chabriere@univ-amu.fr

Supplementary Figures and Tables

Supplementary Data 1 Proteins detected by proteomic analysis, raw data, fold change and functional classification (See excel file: Supplementary_Data_1-ProteomicData)

Supplementary Table 1 Metabolome analysis filtration criteria

Mzmine 2

Step	Parameter	Value		
Mass detection	Mass detector	Centroid		
	Noise level	5,00E+02		
Chromatogram builder	Min group size in # of scans	5		
	Group intensity threshold	5,00E+02		
	Min height	5,00E+02		
	M/z tolerance (absolute)	0.016 / 20ppm		
chromatograme deconvolution	Algorithm	Baseline cut-off		
	Min peak height	1,00E+03		
	Peak duration range (min)	0.005-1		
	baseline level	100		
Chromatogram deisotoped	M/z tolerance (absolute)	0,016		
	Retention time tolerance	0,1		
	Maximum charge	3		
	Representative isotope	Most intense		
Duplicate peak filter	Filter mode	NEW AVERAGE		
	M/z tolerance (absolute)	0.1/20ppm		
	Retention time tolerance (min)	0,3		
Retention time normalizer	M/z tolerance (absolute)	0,016		
	Retention time tolerance (min)	0,3		
	Minimum standard intensity	10000		
Peak list aligned	M/z tolerance (absolute)	0,016		
	Retention time tolerance (min)	0,5		
	Weight for m/z	1	List	8496
	Weight for rt	1		
Gap filled	Intensity tolerance	0,5		
	M/z tolerance (absolute)	0,016		
	Retention time tolerance (min)	0,3		
Duplicate peak filter	Filter mode	NEW AVERAGE		
	M/z tolerance (absolute)	0,2	Matrix	7764
	Retention time tolerance (min)	0,8		
Window delimitation	M/z	100-1500		
	rt	0.5-20	Matrix	7529

Filtration in R

Elimination noise signal	**signal/noise ratio**	< 3	variables 996	
Elimination of non stable signals	**Coefficient of variation**	> 50 %	variables 489	
Manual filtration		-83	variables 406	Matrix used

Metaboanalyst analysis

Data filtering	**Standard deviation (SD)**		
Sample Normalisation	**None**		
Data transformation	**Log transformation**		
Data scaling	**Mean centring**		
Validation du modèle	**Pemutation**	<0.01	
	**R2**	0,93802	Validated model
	**Q2**	0,84125	

Metabolome annotation (molecular networks)

Precursor ion mass tolerance	0,02
Fragment ion mass tolerence	0,02
Min pairs Cos	0,7
Minimum matched fragments ions	4

BDD	**mzmax**
	**rtmax**
Clusters	**≥ 4 metabolites**
	**with 1 annotated node**
Nodes	**Total**
	**Annotated**

Supplementary Table 2 List of VIP metabolites and proposed identification.

VIP N°	*m/z*	RT (min)	VIP score	Molecular formula	Mass error (ppm)	m*σ*[a]	MS/MS fragment ions (relative abundance in %)	Putative annotation	Heatmap: No enzyme	Heatmap: *Sso*Pox W263I
1	240,0690	7.66	3,14	$C_{11}H_{14}NO_3S^+$	-0.2	7.6	192.0656 $[C_{10}H_{10}NO_3]^+$ (7), 174.0544 $[C_{10}H_8NO_2]^+$ (41), 146.0598 $[C_9H_8NO]^+$ (67), 120.0443 $[C_7H_6NO]^+$ (100), 94.0652 $[C_6H_8N]^+$ (42)	Thiobutacin	-5,02	5,02
2	386.1137	7.73	2,97	$C_{22}H_{13}N_3O_4^+$	-0.4	2.2	368.1026 $[C_{22}H_{14}N_3O_3]^+$ (6), 350.0921 $[C_{22}H_{12}N_3O_2]^+$ (50), 324.1123 $[C_{21}H_{14}N_3O]^+$ (100), 296.1167 $[C_{20}H_{14}N_3]^+$ (4)	Chromopyrrolic acid	-4,74	4,74
3	478.2081	8.45	2,92	$C_{25}H_{28}N_5O_5^+$	-1.1	1.5	335.1497 $[C_{19}H_{19}N_4O_2]^+$ (100), 268.0714 $[C_{14}H_{10}N_3O_3]^+$ (62), 231.1242 $[C_{12}H_{15}N_4O]^+$ (6), 212.1280 $[C_{11}H_{18}NO_3]^+$ (4), 116.0707 $[C_5H_{10}NO_2]^+$ (16), 105.0335 $[C_7H_5O]^+$ (20), 89.0597 $[C_4H_9O_2]^+$ (11), 59.0495 $[C_3H_7O]^+$ (10)	?[c]	-4,66	4,66
4	270.1703	6.94	2,84	$C_{14}H_{24}NO_4^+$	-1.1	45.6	252.1608 $[C_{14}H_{22}NO_3]^+$ (2), 169.1222 $[C_{10}H_{17}O_2]^+$ (2),102.0550 $[C_4H_8NO_2]^+$ (11), 74.0601 $[C_3H_8NO]$+ (3), 56.0494 $[C_3H_6N]^+$ (4)	3-oxo-C10-HSL	4,54	-4,54
5	272.1854	6.85	2,74	$C_{14}H_{26}NO_4^+$	1	3.7	254.1750 $[C_{14}H_{24}NO_3]^+$ (In source fragmentation), 236.1639 $[C_{14}H_{22}NO_2]^+$ (5), 153.1266 $[C_{10}H_{17}O]^+$ (40), 135.1166 $[C_{10}H_{15}]^+$ (100), 102.0549 $[C_4H_8NO_2]^+$ (84), 74.0598 $[C_3H_8NO]^+$ (16), 56.0494 $[C_3H_6N]^+$ (6)	3-OH-C10-HSL	4,37	-4,37
6	756.3386	10.64	2,69	$C_{43}H_{50}NO_{11}^+$	-1.0	6.1	450.1557 $[C_{25}H_{24}NO_7]^+$ (24), 432.144 $[C_{25}H_{22}NO_6]^+$ (19), 363.1952 $[C_{24}H_{27}O_3]^+$ (30), 313.1068 $[C_{18}H_{17}O_5]^+$ (38), 283.096 $[C_{17}H_{15}O_4]^+$ (32), 248.0915 $[C_{13}H_{14}NO_4]^+$ (53), 203.0707 $[C_{12}H_{11}O_3]^+$ (82), 138.0551 $[C_7H_8NO_2]^+$ (68), 111.0443 $[C_6H_7O_2]^+$ (93), 89.0598 $[C_4H_9O_2]^+$ (100)	?[d]	4,29	-4,29
7	343.2837	10.35	2,56	$C_{20}H_{39}O_4^+$	1.6	4.6	325.2728 $[C_{20}H_{37}O_3]^+$ (23), 251.2361 $[C_{17}H_{31}O]^+$ (38), 233.2254 $[C_{17}H_{29}]^+$ (50)	Monoacylglycerol (C17:1)	-4,09	4,09
8	224.1286	1.04	2,50	$C_{12}H_{18}NO_3^+$	0.2	8.8	n.f.[e]	Deacetylanisomycin	3,99	-3,99
9	280.1544	5.86	2,50	$C_{15}H_{22}NO_4^+$	0.7	-2.3	224.1276 $[C_{12}H_{18}NO_3]^+$ (8), 206.1174 $[C_{12}H_{16}NO_2]^+$ (36), 188.1067 $[C_{12}H_{14}NO]^+$ (8), 159.0807 $[C_{11}H_{11}O]^+$ (9), 121.0648 $[C_8H_9O]^+$ (100)	Anisomycin propionate derivative	3,99	-3,99
10	286.2012	7.38	2,40	$C_{15}H_{28}NO_4^+$	0.1	6.9	268.1907 $[C_{15}H_{26}NO_3]^+$ (In source fragmentation), 250.1772 $[C_{15}H_{24}NO_2]$+ (3), 167.1418 $[C_{11}H_{19}O]^+$ (17), 149.1325 $[C_{11}H_{17}]^+$ (69), 102.0549 $[C_4H_8NO_2]^+$ (100), 74.0601 $[C_3H_8NO]^+$ (17), 56.0491 $[C_3H_6N]$+ (3)	3-OH-C11-HSL	3,83	-3,83
11	292.1528	6.98	2,34	$C_{16}H_{22}NO_4^+$	5.3	16.2	n.f.	?	3,73	-3,73
12	596.2133	8.56	2,29	$C_{31}H_{34}NO_{11}^+$	-1.2	7.1	450.1557 $[C_{25}H_{24}NO_7]^+$ (17), 432.144 $[C_{25}H_{22}NO_6]^+$ (17), 363.1952 $[C_{24}H_{27}O_3]^+$ (30), 313.1066 $[C_{18}H_{17}O_5]^+$ (26), 283.096 $[C_{17}H_{15}O_4]^+$ (51), 248.0915 $[C_{13}H_{14}NO_4]^+$ (53), 203.0701 $[C_{12}H_{11}O_3]^+$ (81), 138.0551 $[C_7H_8NO_2]^+$ (68), 111.0443 $[C_6H_7O_2]^+$ (93), 89.0598 $[C_4H_9O_2]^+$ (100)	?[d]	3,65	-3,65
13	318.2272	7.25	2,26	$C_{16}H_{32}NO_5^+$	1.0	5.4	n.f.	?	3,60	-3,60
14	266.1385	3.82	1,89	$C_{14}H_{20}NO_4^+$	0.6	4.7	224.1273 $[C_{12}H_{18}NO_3]^+$ (4), 206.1175 $[C_{12}H_{16}NO_2]^+$ (32), 188.1075 $[C_{12}H_{14}NO]^+$ (11), 159.0805 $[C_{11}H_{11}O]^+$ (7), 121.0647 $[C_8H_9O]^+$ (100)	Anisomycin[f]	3,02	-3,02
15	237.2212	10.07	1,85	$C_{16}H_{29}O^+$	0.2	1.7	n.f.	?	-2,96	2,96
16	408.2967	6.86	1,85	$C_{20}H_{42}NO_7^+$	-2.7	5.6	n.f.	?	2,96	-2,95
17	274,2014	8.12	1,83	$C_{14}H_{28}NO_4^+$	-0.3	4.8	256.1905 $[C_{14}H_{26}NO_3]^+$ (6), 238.1822 $[C_{14}H_{24}NO_2]^+$ (1), 155.1434 $[C_{10}H_{19}O]^+$ (6), 137.1326 $[C_{10}H_{17}]^+$ (2), 120.0657 $[C_4H_{10}NO_3]^+$ (100), 102.0551 $[C_4H_8NO_2]^+$ (28), 74.0602 $[C_3H_8NO]^+$ (85), 56.0492 $[C_3H_6N]$+ (11)	C10-HS (Homoserine)	-2,91	2,91
18	298,2020	8.05	1,80	$C_{16}H_{28}NO_4^+$	-0.8	16.7	n.f.	3-oxo-C12-HSL	2,87	-2,87
19	457.1655	7.81	1,75	$C_{29}H_{21}N_4O_2^+$	1.0	4.6	427.1511 $[C_{28}H_{19}N_4O]^+$ (1), 328.108 $[C_{20}H_{14}N_3O_2]^+$ (2), 213.0659 $[C_{12}H_9N_2O_2]^+$ (1), 145.1217 $[C_8H_{17}O_2]$+ (2), 101.0595 $[C_5H_9O_2]^+$ (16), 89.0598 $[C_4H_9O_2]^+$ (13), 59.0493 $[C_3H_7O]^+$ (7)	?	2,79	-2,79
20	549.2707	6.56	1,74	$C_{30}H_{37}N_4O_6^+$	0.1	5.0	275.1387 $[C_{15}H_{19}N_2O_3]^+$ (100), 247.1435 $[C_{14}H_{19}N_2O_2]^+$ (2), 150.0914 $[C_9H_{12}NO]^+$ (7), 121.065 $[C_8H_9O]^+$ (6), 70.0651 $[C_4H_8N]^+$ (2)	?	2,78	-2,78
21	517.2202	8.29	1,52	$C_{27}H_{29}N_6O_5^+$	-1.7	11.8	374.1608 $[C_{21}H_{20}N_5O_2]^+$ (100), 307.0819 $[C_{16}H_{11}N_4O_3]^+$ (4), 275.1141 $[C_{13}H_{15}N_4O_3]^+$ (4), 147.1009 $[C_7H_{15}O_3]^+$ (2), 133.0857 $[C_6H_{13}O_3]^+$ (4), 103.039 $[C_7H_{11}O_3]^+$ (3), 89.0597 $[C_4H_9O_2]^+$ (7), 59.0493 $[C_3H_7O]^+$ (4)	?[c]	-2,40	2,40

[a] Constructor statistical match factor (comparison of theoretical and experimental isotopic patterns). [b] n.d. : Not determined. [c] Vips n°4 and n°24 showed a similar MS/MS fragmentation pattern. [d] Vips n°8 and n°15 showed similar MS/MS fragment ions. [e] n.f. : Not fragmented. [f] Identification confirmed using a commercial standard.

Normalized scaled intensity	-6	-3	0	3	6
Color code	-6	-3	0	3	6

Supplementary Table 3. Kinetic parameters of *Sso*Pox W263I for C9-HSL, C10-HSL, C11-HSL, C12-HSL, 3-OH-C10-HSL, 3-oxo-C10-HSL and 3-oxo-C12-HSL.

**AHLs**	**C9-HSL**	**C10-HSL**	**C11-HSL**	**C12-HSL**	**3-OH-C10-HSL**	**3-oxo-C10-HSL**	**3-oxo-C12-HSL**
**k_{cat} (10^{-1} s^{-1})**	1.52 ± 0.07	0.90 ± 0.13	0.70 ± 0.12	ND	1.03 ± 0.04	6.00 ± 0.90	18.0 ± 0.5
**K_M (μM)**	347 ± 46	664 ± 208	1030 ± 340	ND	491 ± 48	1605 ± 443	17.8 ± 4.9
**k_{cat}/K_M ($M^{-1}.s^{-1}$)**	439 ± 154	135 ± 60	68 ± 35	ND	210 ± 83	374 ± 117	(101 ± 28) × 10^3
**Results from**	This study	This study	This study	This study	This study	[1]	[1]

ND: not detected

1. Hiblot, J., Gotthard, G., Elias, M. & Chabriere, E. Differential Active Site Loop Conformations Mediate Promiscuous Activities in the Lactonase SsoPox. *PLoS One* **8**, e75272 (2013).

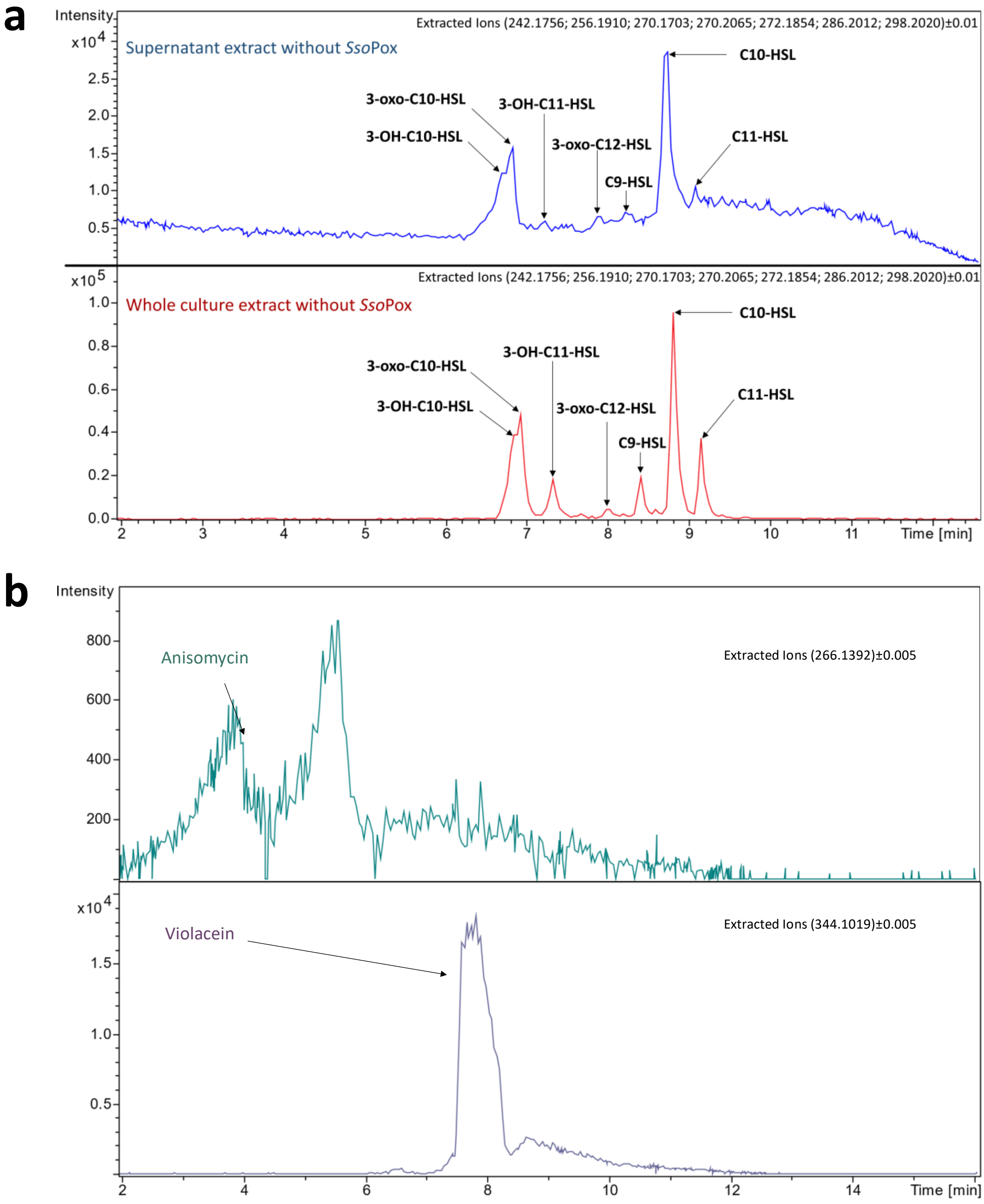

Supplementary Figure 1. LC-MS extracted ion chromatograms of supernatant and whole culture extracts of cultures of *C. violaceum* not treated with *Sso*Pox. (a) Identification of HSL in whole culture and supernatant extracts. **(b)** Identification of anisomycin and violacein in supernatant extracts.

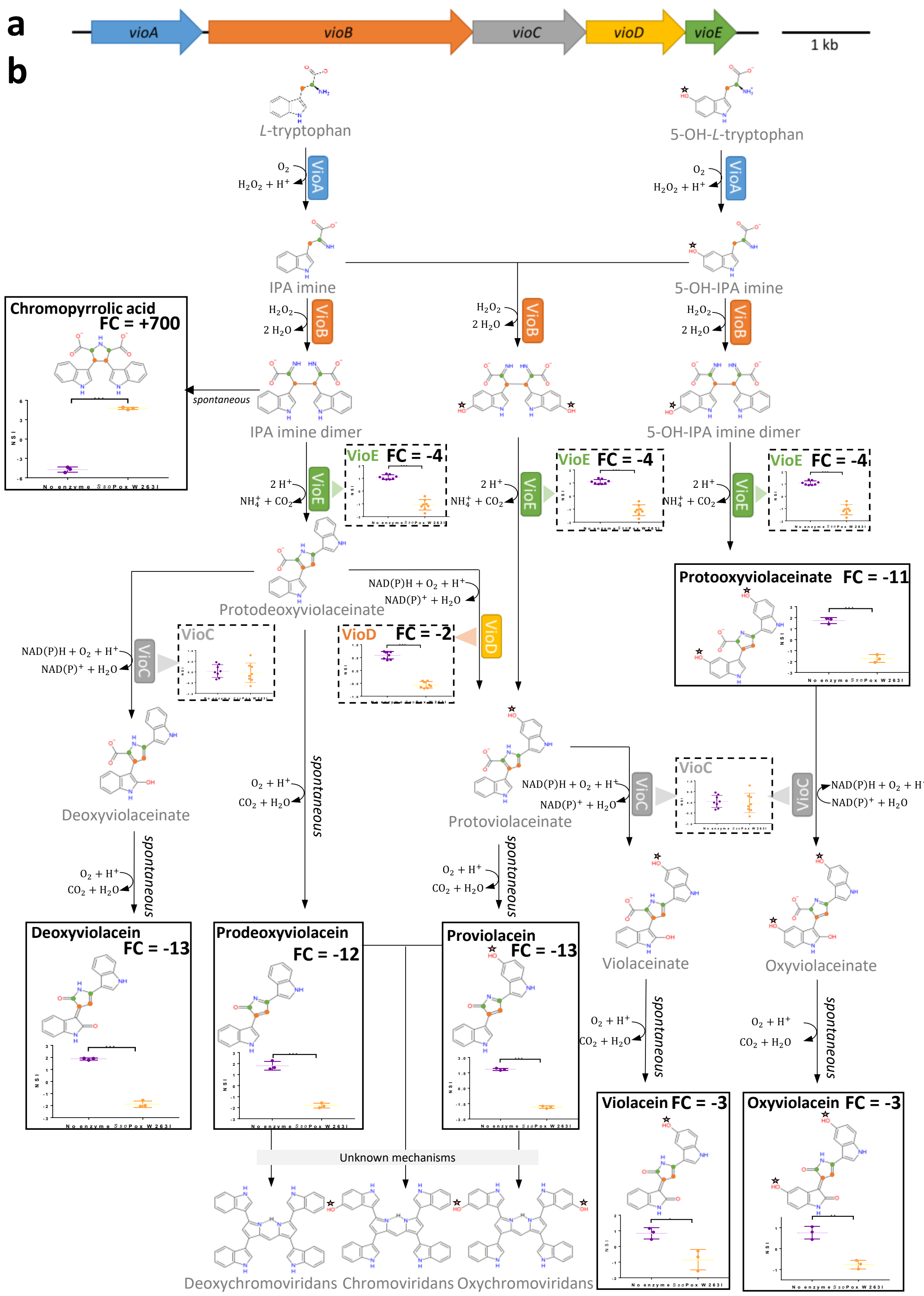

Supplementary Figure 2. Impact of *Sso*Pox W263I on violacein biosynthesis in *C. violaceum* - extended figure. (**a**) Organization of the violacein biosynthesis operon. Arrows indicate direction of transcription. (**b**) Representation of *Sso*Pox W263I impact on violacein biosynthesis by CV 12472 untreated (purple) and treated cultures (yellow). Proteins (dashed line boxes) and metabolites (full line boxes) normalized scaled intensities (NSI) are represented as the mean and standard deviations (metabolomic: $n = 3$ biological replicates, proteomic: $n = 8$, 4 biological replicates × 2 technical replicates). *p-value < 0.05, **p-value < 0.01, ***p-value < 0.001 according to Student's t-test. FC: fold change.

a

Chromobacterium violaceum ATCC 12472				*Streptomyces hygrospinosus* ACCC40033					
Gene	Uniprot	Size, aa	Predicted function	Gene	Size, aa	Predicted function	E-value	Query cover	Identities
CV_2255	Q7NVT7	173	Maltose O-acetyltransferase	*aniI*	222	O-acetyltransferase	1E-32	92%	32%
CV_2256	Q7NVT6	412	Acetylornithine aminotranfserase ArgD	*aniQ*	441	PLP-dependent aminotransferase	4E-81	94%	35%
CV_2257	Q7NVT5	555	Transketolase domain-containing protein	*aniP*	603	Transketolase	4E-84	94%	35%
CV_2258	Q7NVT4	281	Probable short-chain dehydrogenase	*aniN*	275	NAD(P)-dependent short-chain dehydrogenase	1E-76	95%	43%
CV_2259	Q7NVT3	198	Uncharacterized protein	*aniM*	208	Hypothetical protein	2E-25	93%	36%
CV_2260	Q7NVT2	261	S6PP domain-containing protein	*aniH*	255	Hypothetical protein	0.0005	62%	24%
CV_2261	Q7NVT1	341	Probable acetylserotonin O-methyltransferase	*aniK*	331	SAM-dependent O-methyltransferase	8E-31	96%	28%
CV_2262	Q7NVT0	434	MFS transporter	*aniL*	393	MFS transporter	1.7	18%	33%
				aniO	462	Glycosyltransferase			
				aniG	577	α-glucosidase			
				aniF	208	LuxR family transcriptional regulator			
				aniJ	291	Putative phosphatase			

b

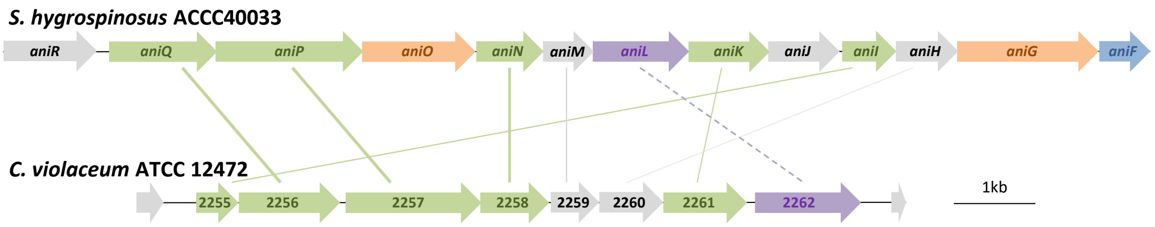

Supplementary Figure 3. Identification of anisomycin biosynthesis gene cluster in *C. violaceum* ATCC 12472 and comparison with *Streptomyces hygrospinosus* ACCC40033. (**a**) Blast comparisons of proteins involved in anisomycin biosynthesis between *C. violaceum* and *S. hygrospinosus*. Genes written in green have been previously shown to be necessary for the full anisomycin biosynthesis from *L*-tyrosine in *S. hygrospinosus*; genes written in orange are those being involved in the glycosylation and deglycosylation steps of anisomycin and its intermediates; genes written in grey have not been shown as being involved in the anisomycin biosynthesis; aniF (blue) is involved in the anisomycin regulation and aniL (purple) codes for a transporter 1. (**b**) Comparison of *S. hygrospinosus* and *C. violaceum* gene cluster organizations. Full lines indicate sequence identities as revealed by Blast analyses and thickness of the lines represents E-values. The dashed line indicates a similar function without any sequence homology.

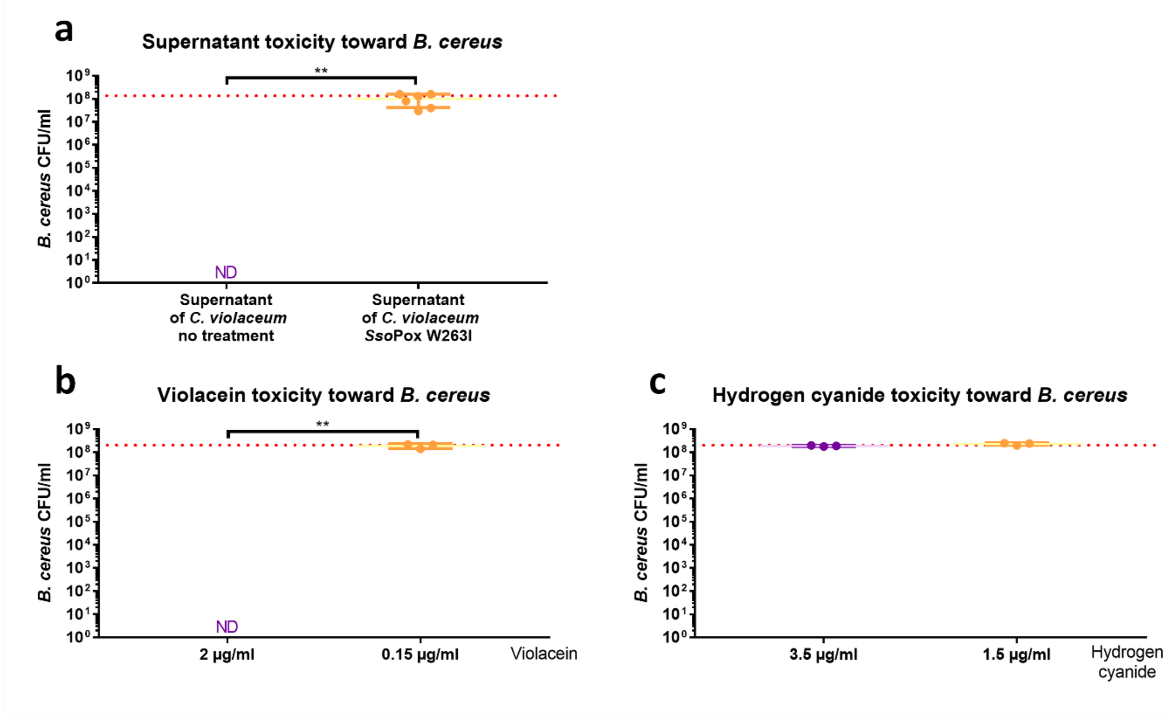

Supplementary Figure 4. Effect of *C. violaceum* supernatant, violacein and hydrogen cyanide on the growth of *B. cereus*. Mean bacterial concentration in CFU.ml^{-1} of *B. cereus* after 8 hours of culture in the presence of 50% of supernatant from treated cultures of *C. violaceum* with 0.5 mg ml^{-1} *Sso*Pox W263I (yellow bar) or untreated cultures as negative controls (purple bar) (**a**) and in the presence of violacein (**b**) and hydrogen cyanide (**c**) in quantity equivalent to the one in 50% of supernatant from treated cultures of *C. violaceum* with 0.5 mg ml^{-1} *Sso*Pox W263I (yellow bar) or in untreated cultures (purple bar). Error bars represent the standard deviations of *n* = 6 experiments (3 biological replicates × 2 technical replicates) or *n* = 3 biological replicates, each represented by one point. ***p*-values < 0.01 according to Student's *t*-test. ND: Not detected. Dotted red line represents the mean bacterial concentration of *B. cereus* with 50% of culture medium alone.

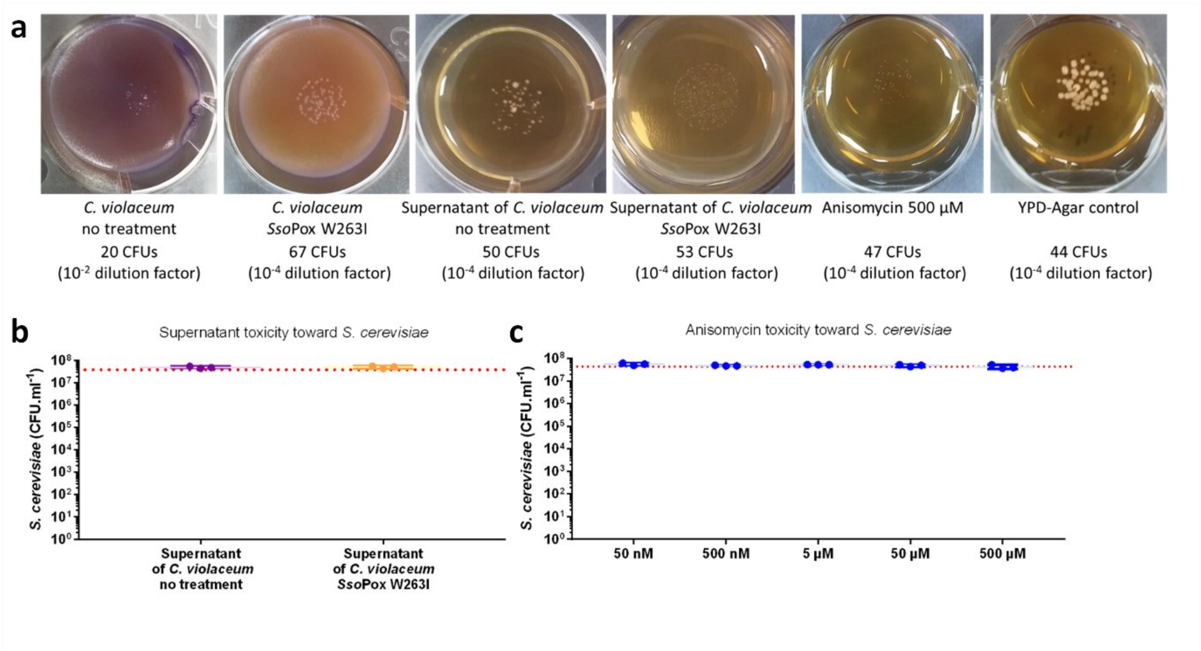

Supplementary Figure 5. *C. violaceum* and anisomycin impact on colony size and growth of *S. cerevisiae.* (a) Representative pictures of colony formation of *S. cerevisiae* after 24 h at 30°C from left to right : on YPD-Agar infected by untreated *C. violaceum* (dilution factor at 10^{-2}), on YPD-Agar infected by *C. violaceum* treated with 0.5 mg.ml^{-1} *Sso*Pox W263I (dilution factor at 10^{-4}), on YPD-Agar containing 50% of supernatant of untreated *C. violaceum* (dilution factor at 10^{-4}), on YPD-Agar containing 50% of supernatant of *C. violaceum* treated with 0.5 mg.ml^{-1} *Sso*Pox W263I (dilution factor at 10^{-4}), on YPD-Agar containing 500 μM of anisomycin (dilution factor at 10^{-4}), on YPD-Agar for control (dilution factor at 10^{-4}). Pictures are representative of n = 3 independent replicates. (**b**) Mean yeast concentration in CFU.ml^{-1} of *S. cerevisiae* after 24 hours growth on YPD-agar containing 50% of supernatant from treated cultures of *C. violaceum* with 0.5 mg ml^{-1} *Sso*Pox W263I (yellow bar) or untreated cultures as negative controls supernatant (purple bar). Error bars represent the standard deviations of *n* = 3 biological replicates. Dotted red line represents the mean yeast concentration of *S. cerevisiae* on YPD-Agar only. (**c**) Mean yeast concentration in CFU.ml^{-1} of *S. cerevisiae* after 24 hours growth on YPD-agar containing anisomycin at concentrations ranging from 50 nM to 500 μM. Error bars represent the standard deviations of *n* = 3 biological replicates. Dotted red line represents the mean yeast concentration of *S. cerevisiae* on YPD-Agar only.

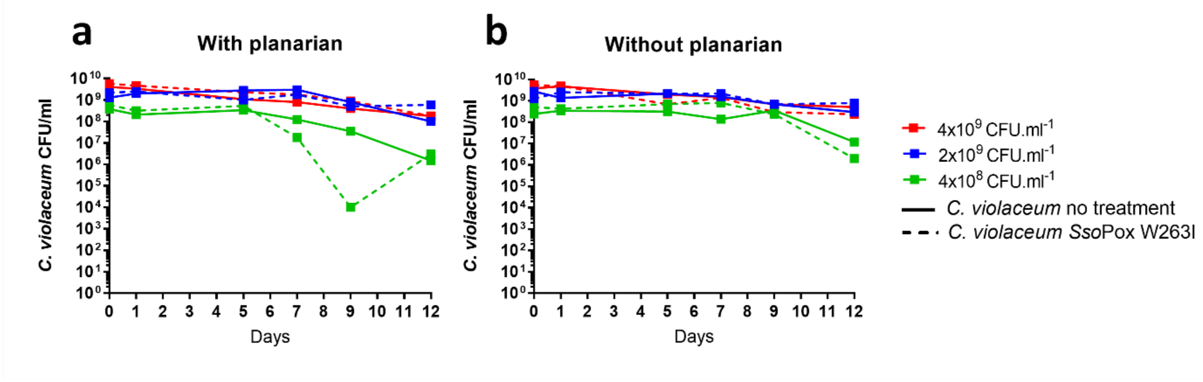

Supplementary Figure 6. Evolution of bacterial concentration of *C. violaceum* with and without planarian over time. Bacterial concentration in CFU.ml^{-1} of *C. violaceum* treated with 0.5 mg.ml^{-1} *Sso*Pox W263I (full line) and untreated cultures (dotted line) in well containing planarians (**a**) and well without worm (**b**).

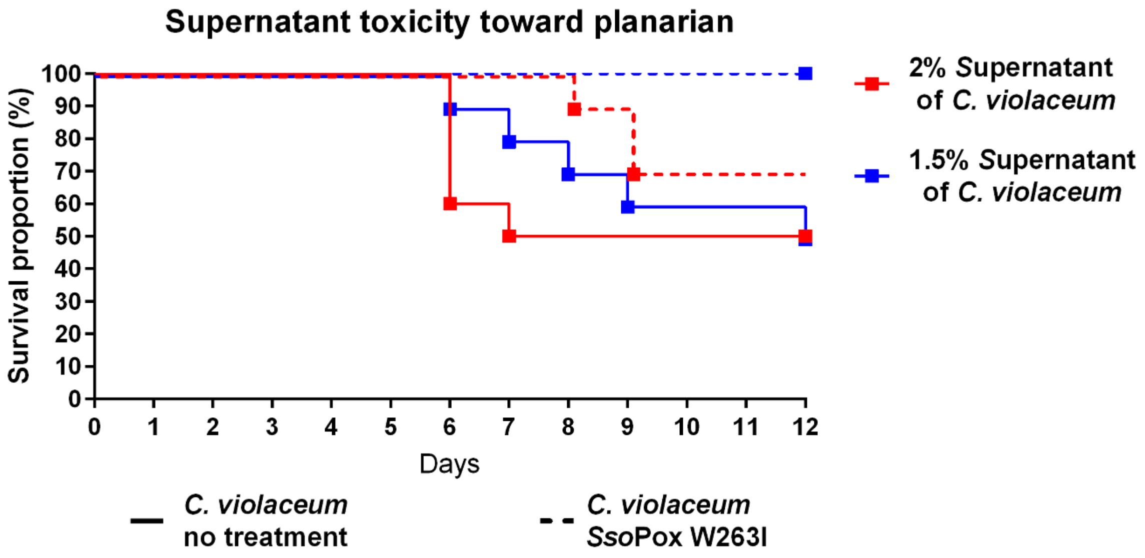

Supplementary Figure 7. Planarian survival in the presence of supernatants of *C. violaceum* treated with 0.5 mg.ml^{-1} *Sso*Pox W263I (dotted line) and untreated cultures as negative controls (full line) at 2% (v/v in tap water) (red) and 1.5% (v/v in tap water) (blue). Curves represent survival proportions of 10 planarians. According to log-rank (Mantel-Cox) test comparing survival curves in treated and untreated conditions p-value = 0.2294 for 2% of supernatant and p-value = 0.0115 for 1.5% of supernatant.

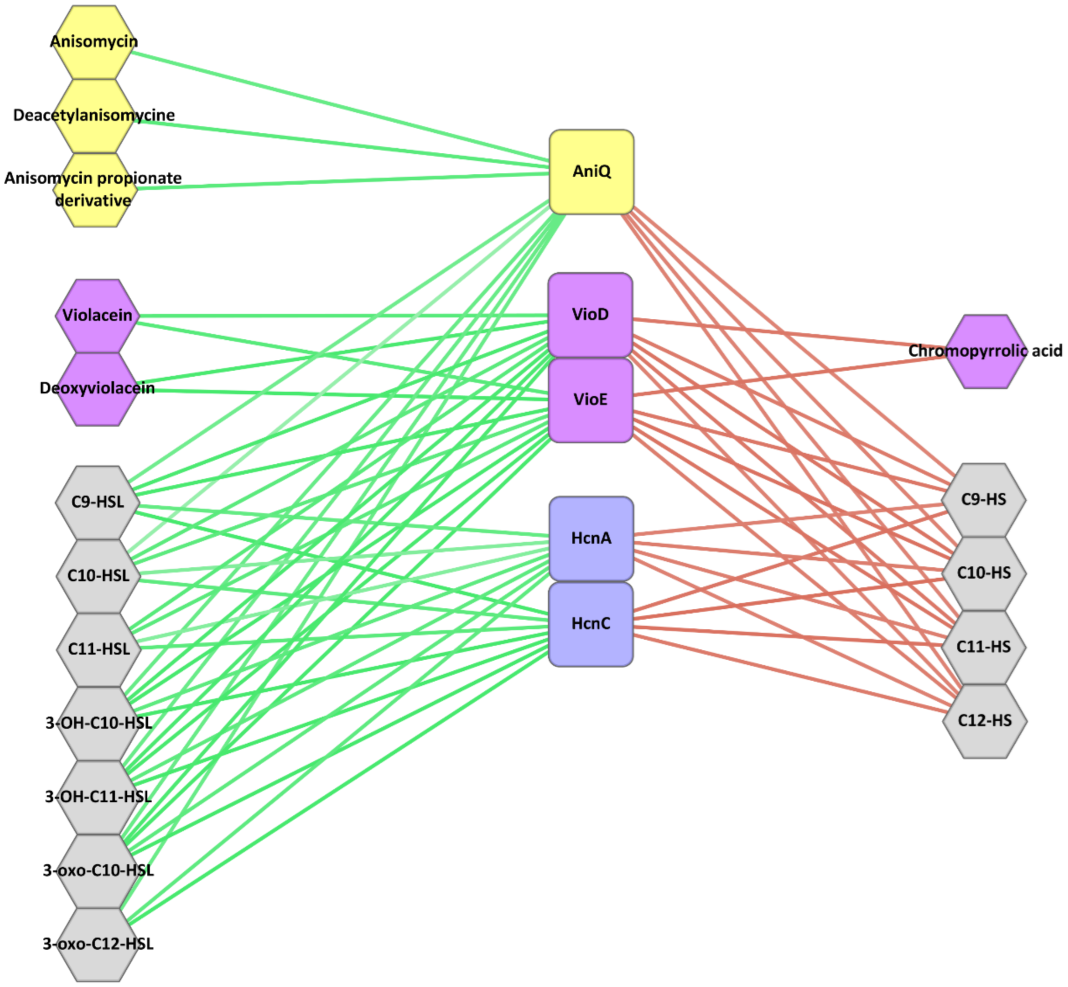

Supplementary Figure 8. *Sso*Pox impacts jointly the biosynthesis of toxic metabolites and the production of AHLs. Multi-omics correlation network between AHLs (grey), their hydrolyzed form HSs (grey) and molecules involved in anisomycin (yellow), violacein (purple) and hydrogen cyanide (blue) production.
