# Supplementary material for: Disrupting quorum sensing alters social interactions in Chromobacterium violaceum
Source: NPJ Biofilms Microbiomes. 2021 Apr 22;7:40. doi: 10.1038/s41522-021-00211-w (PMC8062528; doi:10.1038/s41522-021-00211-w)
Supplement: Supplementary file 3 — Reporting Summary [file 41522_2021_211_MOESM3_ESM.pdf]

nature research

Corresponding author(s): *Double-blind peer review submissions: write DBPR and your manuscript number here instead of author names.*

Last updated by author(s): *YYYY-MM-DD*

Reporting Summary

Nature Research wishes to improve the reproducibility of the work that we publish. This form provides structure for consistency and transparency in reporting. For further information on Nature Research policies, see our Editorial Policies and the Editorial Policy Checklist.

Statistics

For all statistical analyses, confirm that the following items are present in the figure legend, table legend, main text, or Methods section.

n/a	Confirmed	
☐	☒	The exact sample size (*n*) for each experimental group/condition, given as a discrete number and unit of measurement
☐	☒	A statement on whether measurements were taken from distinct samples or whether the same sample was measured repeatedly
☐	☒	The statistical test(s) used AND whether they are one- or two-sided<br>*Only common tests should be described solely by name; describe more complex techniques in the Methods section.*
☒	☐	A description of all covariates tested
☒	☐	A description of any assumptions or corrections, such as tests of normality and adjustment for multiple comparisons
☐	☒	A full description of the statistical parameters including central tendency (e.g. means) or other basic estimates (e.g. regression coefficient) AND variation (e.g. standard deviation) or associated estimates of uncertainty (e.g. confidence intervals)
☒	☐	For null hypothesis testing, the test statistic (e.g. *F*, *t*, *r*) with confidence intervals, effect sizes, degrees of freedom and *P* value noted<br>*Give P values as exact values whenever suitable.*
☒	☐	For Bayesian analysis, information on the choice of priors and Markov chain Monte Carlo settings
☒	☐	For hierarchical and complex designs, identification of the appropriate level for tests and full reporting of outcomes
☒	☐	Estimates of effect sizes (e.g. Cohen's *d*, Pearson's *r*), indicating how they were calculated

Our web collection on statistics for biologists contains articles on many of the points above.

Software and code

Policy information about availability of computer code

Data collection	*Provide a description of all commercial, open source and custom code used to collect the data in this study, specifying the version used OR state that no software was used.*
Data analysis	*Provide a description of all commercial, open source and custom code used to analyse the data in this study, specifying the version used OR state that no software was used.*

For manuscripts utilizing custom algorithms or software that are central to the research but not yet described in published literature, software must be made available to editors and reviewers. We strongly encourage code deposition in a community repository (e.g. GitHub). See the Nature Research guidelines for submitting code & software for further information.

Data

Policy information about availability of data

All manuscripts must include a data availability statement. This statement should provide the following information, where applicable:
- Accession codes, unique identifiers, or web links for publicly available datasets
- A list of figures that have associated raw data
- A description of any restrictions on data availability

All data have been used in this paper and raw data are available upon request.

Field-specific reporting

Please select the one below that is the best fit for your research. If you are not sure, read the appropriate sections before making your selection.

☐ Life sciences ☐ Behavioural & social sciences ☒ Ecological, evolutionary & environmental sciences

For a reference copy of the document with all sections, see nature.com/documents/nr-reporting-summary-flat.pdf

Ecological, evolutionary & environmental sciences study design

All studies must disclose on these points even when the disclosure is negative.

Study description	Using combined metabolomic and proteomic approaches, we demonstrated that QS and enzyme-based QQ modulate the production of antimicrobial and toxic compounds in C. violaceum ATCC 12472. These modifications resulted in drastic changes in social interactions between C. violaceum and a Gram-positive bacterium (Bacillus cereus), a yeast (Saccharomyces cerevisiae), immune cells (murine macrophages) and an animal model (planarian Schmidtea mediterranea).
Research sample	Model laboratory strains and organisms were used in this study.<br>Chromobacterium violaceum ATCC 12472, Bacillus cereus ATCC 1457 and Saccharomyces cerevisiae ATCC 9763 are standard microbiological strains.<br>J774.1 macrophages (murine macrophage) are standard eukaryotic cell lines.<br>Freshwater planarians belonging to the Schmidtea mediterranea species (asexual clonal line ClW4) were used for the experiment and constitute a research animal model that does not require ethical approval.
Sampling strategy	Sampling strategy was designed according to standard laboratory procedures.<br>For proteomics analysis, four samples were prepared for each condition and injected twice.<br>For metabolomic analysis, three replicates per condition were prepared along with 6 media blanks and 4 QC samples.<br>Microbiological assays (violacein, HCN, protease and biofilm) were performed in triplicates<br>Competition assays (Bacillus cereus, Saccharomyces cerevisiae, and murine macrophages) were performed in triplicates.<br>Animal testing with planarians were performed in triplicates with 10 worms for each condition.
Data collection	Data were collected by experimentators according to normal procedures.
Timing and spatial scale	Experiments spread from june 2018 to february 2021.<br>All experiments were performed at the IHU of Marseille (France) except metabolomic studies that were performed at MAPIEM laboratory in Toulon (France
Data exclusions	No data were excluded
Reproducibility	All attempts to repeat the experiments were successful. The data presented in the manuscript are representative of all the attempts that were made.
Randomization	Not relevant to our study as no cohort nor clinical trials are involved
Blinding	Not relevant to our study as no cohort nor clinical trials are involved

Did the study involve field work? ☐ Yes ☒ No

Reporting for specific materials, systems and methods

We require information from authors about some types of materials, experimental systems and methods used in many studies. Here, indicate whether each material, system or method listed is relevant to your study. If you are not sure if a list item applies to your research, read the appropriate section before selecting a response.

Materials & experimental systems

n/a	Involved in the study	
☒	☐	Antibodies
☐	☒	Eukaryotic cell lines
☒	☐	Palaeontology and archaeology
☐	☒	Animals and other organisms
☒	☐	Human research participants
☒	☐	Clinical data
☒	☐	Dual use research of concern

Methods

n/a	Involved in the study	
☒	☐	ChIP-seq
☒	☐	Flow cytometry
☒	☐	MRI-based neuroimaging

Eukaryotic cell lines

Policy information about cell lines

Cell line source(s)	murine macrophage
Authentication	J774.1 macrophages
Mycoplasma contamination	Cell lines were not tested for mycoplasma contamination
Commonly misidentified lines (See ICLAC register)	*Name any commonly misidentified cell lines used in the study and provide a rationale for their use.*

Animals and other organisms

Policy information about studies involving animals; ARRIVE guidelines recommended for reporting animal research

Laboratory animals	Freshwater planarians belonging to the Schmidtea mediterranea species (asexual clonal line ClW4) were used for the experiment. The planarians were maintained in autoclaved water at 19 °C in the dark and fed twice a week with calf liver. The animals were starved for at least one week prior to the experiments. The water was changed every two days and did not contain antibiotics. Worms were manually selected to fall within a certain range of size, around 0.8–1 cm in length.
Wild animals	The study did not involve wild animals
Field-collected samples	The study did not involve samples collected from the field
Ethics oversight	No ethical approval for this study as no vertebrates nor mammalians were involved.

Note that full information on the approval of the study protocol must also be provided in the manuscript.
